# Dynamic cues for whisker-based object localization: An analytical solution to vibration during active whisker touch

**DOI:** 10.1371/journal.pcbi.1006032

**Published:** 2018-03-27

**Authors:** Roman Vaxenburg, Isis Wyche, Karel Svoboda, Alexander L. Efros, Samuel Andrew Hires

**Affiliations:** 1 Computational Materials Science Center, George Mason University, Fairfax, Virginia, United States of America; 2 Section of Neurobiology, Department of Biological Sciences, University of South California, Los Angeles, California, United States of America; 3 Janelia Research Campus, Howard Hughes Medical Institute, Ashburn, Virginia, United States of America; 4 Center for Computational Material Science, Code 6390, Naval Research Laboratory, Washington, DC, United States of America; Northwestern University, UNITED STATES

## Abstract

Vibrations are important cues for tactile perception across species. Whisker-based sensation in mice is a powerful model system for investigating mechanisms of tactile perception. However, the role vibration plays in whisker-based sensation remains unsettled, in part due to difficulties in modeling the vibration of whiskers. Here, we develop an analytical approach to calculate the vibrations of whiskers striking objects. We use this approach to quantify vibration forces during active whisker touch at a range of locations along the whisker. The frequency and amplitude of vibrations evoked by contact are strongly dependent on the position of contact along the whisker. The magnitude of vibrational shear force and bending moment is comparable to quasi-static forces. The fundamental vibration frequencies are in a detectable range for mechanoreceptor properties and below the maximum spike rates of primary sensory afferents. These results suggest two dynamic cues exist that rodents can use for object localization: vibration frequency and comparison of vibrational to quasi-static force magnitude. These complement the use of quasi-static force angle as a distance cue, particularly for touches close to the follicle, where whiskers are stiff and force angles hardly change during touch. Our approach also provides a general solution to calculation of whisker vibrations in other sensing tasks.

## Author summary

Vibrations play an important role in the sense of touch in many species, but exactly how they influence touch perception remains mysterious. An important reason for this mystery is the difficulty in measuring vibrations during touch. Mice are a powerful model system for investigating touch perception because they actively sweep their whiskers into objects and the resulting bending from touch can be video recorded. However, vibrations of the whiskers during touch are usually too small and fast to be seen. To overcome this limitation, we develop a new mathematical approach to calculating whisker vibrations from the speed at impact, maximum whisker bending during touch, and location of contact along a whisker, which is more easily observed. We find that vibration frequency and amplitude is strongly dependent on the location of contact along the whisker, which mice may use to deduce the distance between their face and touched objects. We confirm our calculations with high-speed imaging of whisker vibration during touch.

## Introduction

Vibration of tactile sensors contributes to perception of surface texture and object identification in humans [1, 2], prosthetic devices [3, 4], and potentially rodents [5–8]. Vibration could also be an important cue for determining the distance to objects using swept sensors. This method of distance determination is important for mice and rats, who rely on active touch of swept whiskers for navigation and object localization in their natural habitat. It is also important for visually impaired people, who navigate, locate, and identify nearby objects by touch with a swept white cane [9].

When an elastic beam strikes an object, the beam bends and vibrates. The frequencies of this vibration are dependent on where along the beam contact is made [10]. Sensing of vibrational frequency was proposed as a possible method for distance determination using artificial swept antennae [11]. This method has been demonstrated with artificial cylindrical whiskers swept into objects [12] and for similar whiskers held fixed as a textured drum steadily rotates against them [13]. This supports the possibility that rodents could use vibration as a cue for distance to object. However, rodent whiskers are approximately conical [14, 15], with the center of mass one quarter length from the whisker base. This provides conical whiskers with distinct vibrational properties. In addition, the relative lack of mass near the tip of the whisker might make vibrations a less informative cue about object distance during distal contacts.

Since whiskers are conical, they tend to bend much more during distal rather than proximal contacts. This is because the bending stiffness of a beam with a circular cross section is proportional to the fourth power of its radius. For the same push angle (*i*.*e*. the maximum angle the base rotates towards an object during touch), the angle of the force applied by the pole to the follicle is strongly dependent on object distance [16]. This results in different ratios of axial to lateral forces and moments for proximal and distal touches [17], which was proposed as a behavioral basis of radial distance discrimination in head-fixed rodents [18, 19]. In comparison, force magnitude or push angle during touch provides degenerate signals during active radial distance discrimination by mice, neither of which alone predict the behavior of the animal.

Recent biomechanical modeling and experiments on isolated whiskers show that both force angle and vibration can be used to mechanically discriminate radial distance of contact [20]. In rodents, quasi-steady state forces drive activity in slowly adapting and Merkle-cell mechanosensory afferents in the whisker follicle [21, 22], while vibrational dynamics could be well suited for activation of fast-adapting mechanoreceptors [21, 23]. Are the forces and whisker dynamics during actual object localization by head-fixed mice suitable for radial distance discrimination by vibration? Under what conditions would vibration frequency, magnitude, or force angle be a more informative cue? We address these questions by examining quasi-static and dynamic forces evoked by active touch by head-fixed mice locating objects with their whiskers.

We present a new analytical approach to model whisker dynamics generated by contact with objects. This solution builds upon prior work [20, 24]. We experimentally constrain the model by measuring parameters which control these dynamics, including Young’s modulus, damping coefficient, and the temporal dynamics of whisker bending during active touch. We estimate time varying forces applied to the whisker by the object using a quasi-static approximation of whisker bending, then apply the model to calculate vibrational whisker dynamics, bending moment, and shear force at the follicle. The calculated vibrations closely match vibrations we observe under high speed imaging. We find that the vibration frequency during touch provides a unique signature for object distance during contacts along the proximal two thirds of a whisker. Since the proximal half of whiskers are relatively stiff, vibration frequency provides a more sensitive cue for discriminating object distance than the angle of applied force for touches in this range. We also find that the relative magnitude of vibrational forces to quasi-static forces dramatically increases at distal object locations. Thus, neural circuits which compare the relative magnitude of vibrational to sustained forces evoked by touch could provide another distance cue.

## Results

We highlight the key findings in the Results section, with a full derivation contained within the Materials and Methods section.

### Mechanical properties of mouse whiskers during active touch

**Fig 1:**
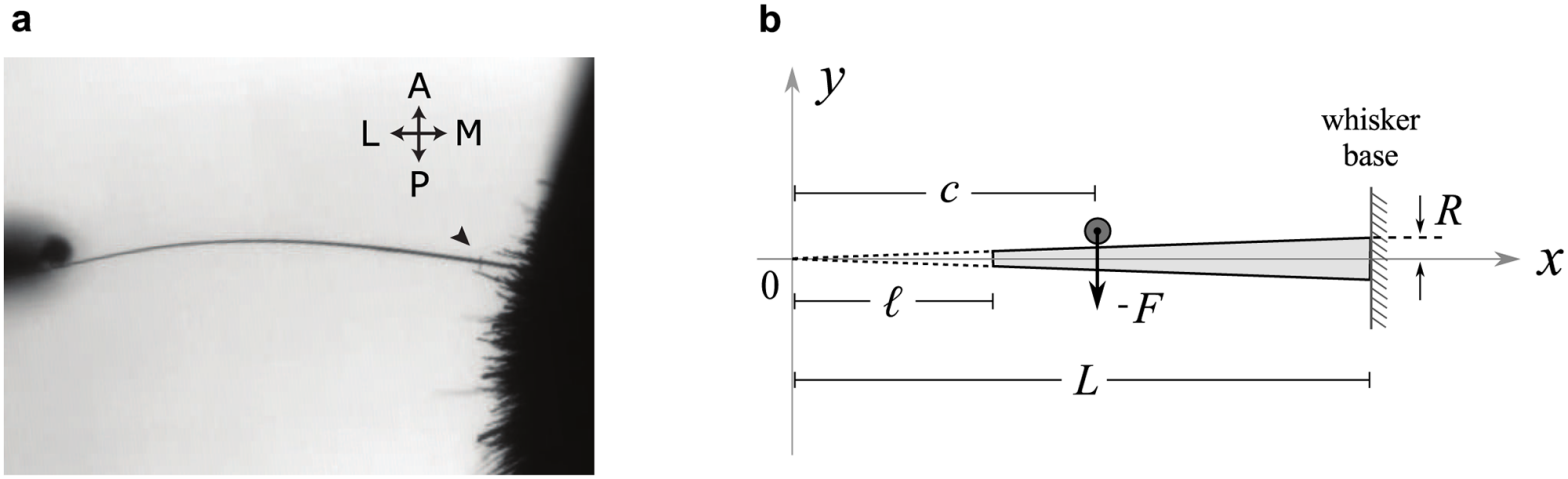
Schematic of whisker touch. A, Video frame with an overhead view of a whisker striking a pole during object localization in head-fixed mice. Arrow points to a dust particle used to track radial movements. Compass indicates anterior-posterior and medial-lateral axes. B, Coordinate frame and configuration of the conical whisker and the pole used in the model. *L* is the length of a full-length whisker, *ℓ* is the truncation length, *R* is the whisker radius at base, and *c* is the position of the pole exerting force *F*. Extrapolated virtual tip of the trimmed whisker is located at *x* = 0.

**Fig 2:**
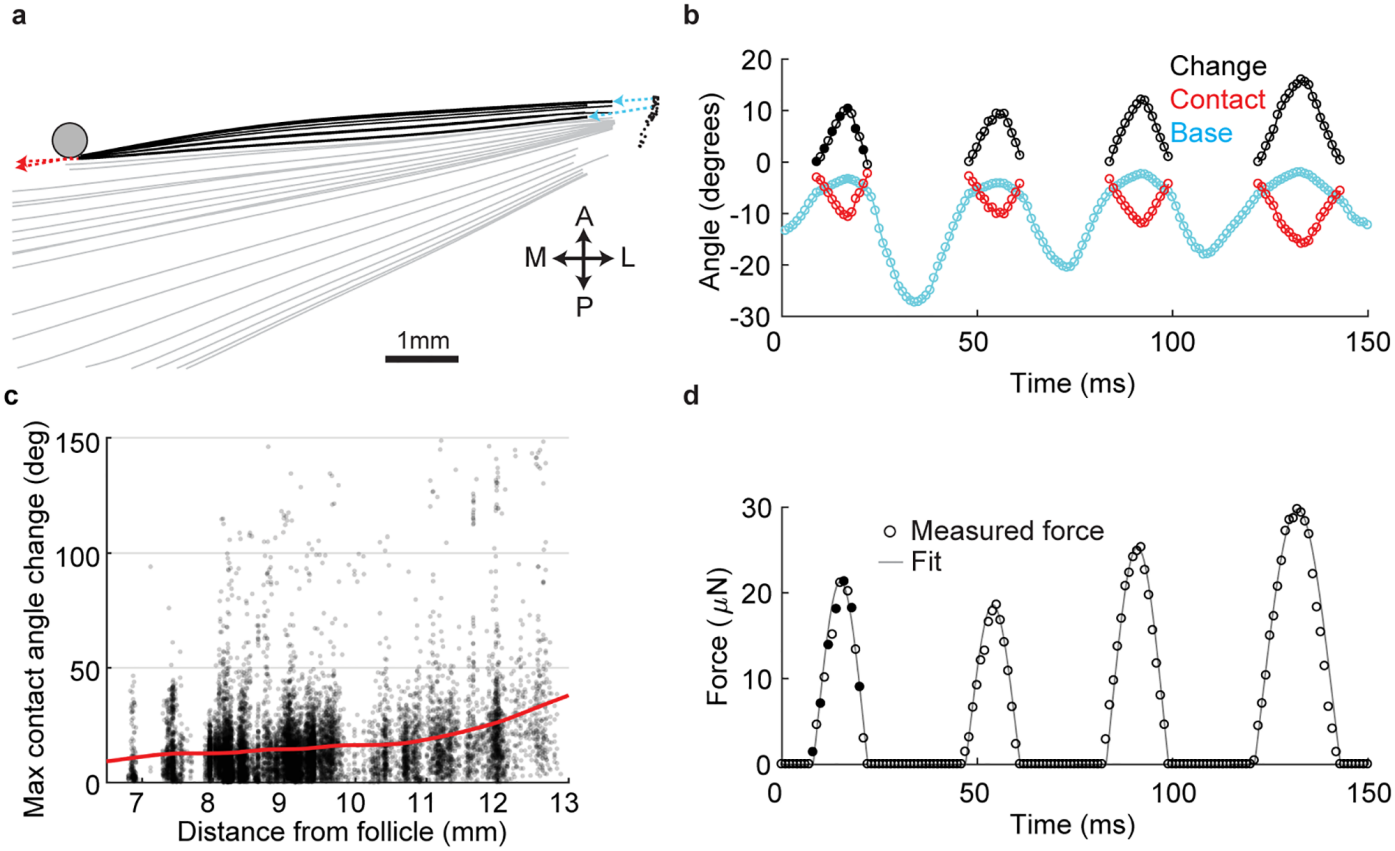
Quantification of active whisker touch. A, Time-series of a tracked whisker before, during and after a typical pole touch. Non-contact periods in gray, contact periods in black. Black dots, estimated base of follicle. Arrows indicate base whisker angle (cyan) and whisker angle at contact point (red). B, Example set of whisker-pole touches. Whisker angle at base (cyan) and at contact point (red). The change in the difference of base angle and contact angle from onset of each touch (black). Time points for traces during contact displayed in (A) are indicated as solid circles. C, Distribution of the maximum change in base-contact angle for 12,361 exploratory (pre-decision) protraction touches across a range of pole distances during object localization. Smoothed spline fit (red). Data from Hires et al. 2015. D, Estimated quasi-static force generated by the four touches in B, using the methodology of Pammer et al., 2013.

We trimmed head-fixed mice to a single whisker (C2) and observed their interactions with a thin vertical pole presented at varying distances from the mouse’s face (6.5–13mm from follicle; Fig 1a). Mouse whiskers are thin tapered elastic beams of roughly conical shape [14], which we describe using the variables illustrated in Fig 1b. Tracking whisker motion and bending from a top-down view at 1000 frames per second revealed that the follicle translates, the whisker bends, and the angle of the whisker at follicle base and angle at object contact change during single touches (Fig 2a and 2b). The difference between base and contact angles defines the angle of the normal force (force angle) and is proportional to the axial (pushing into follicle) and lateral (pushing sideways on the follicle) forces and bending moment (torque applied to follicle). The ratio of axial force to lateral force or bending moment has been proposed to be used by mice to discriminate object distance during contact with a single whisker [18]. Since whiskers become much more flexible near the tip, Weber’s law predicts distance sensing resolution will be maximum near the tip [17].

To test the prediction that force angle changes more dramatically during touches near the whisker tip, we analyzed a set of 12,361 touches from a dataset of head-fixed single whisker object localization [25, 26]. These touches were all active, exploratory touches that informed the animal’s decision about object location, as they all occurred prior to the first lick in a trial. Consistent with the prediction, we found that the maximum change in force angle for each touch was, on average, small at distances <10mm from the follicle (10.3–16.4 deg from 6.7–10mm from base), and increased rapidly near the tip (16.4–36.2 deg from 10–12.7mm) Fig 2c). This suggests that force angle may be a poor discriminator of distance between object positions <10mm from the follicle (about 2/3 of the whisker length). From this whisker bending and estimates shape of the whiskers, we used the a quasi-steady state method to estimate the temporal profile and magnitude of touch forces [16, 18] (Fig 2d).

Tracking dust particles on the whisker (Fig 1a, arrow) during a subset of trials allowed us to determine if whiskers are pushed into the face by axial forces that build up when the whisker bends. The radial location of the follicle moved over 0.6 mm as mice moved their cheek while investigating the pole, and up to 0.2 mm into or out of the pad during single contacts. However, there was no correlation between increasing whisker curvature, which corresponds to increasing axial force, and the radial location of the dust particle. This suggests that the axial position of the follicle is actively controlled, and allows us to neglect axial compliance.

**Fig 3:**
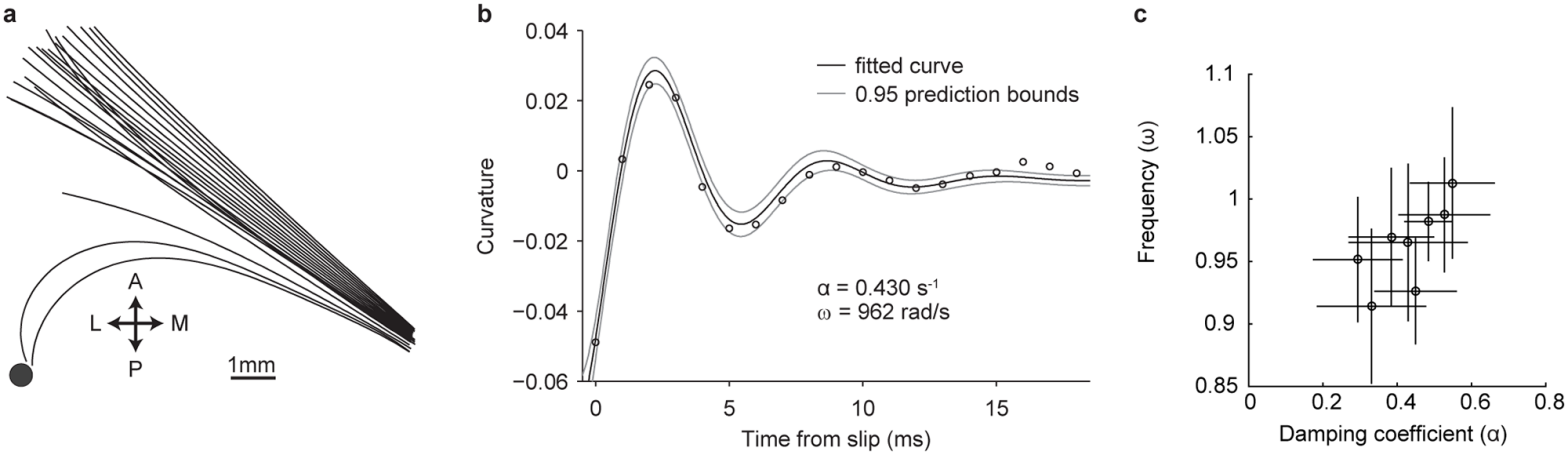
Fitting whisker damping. A, Single tracked whisker over time, immediately before and after a slip-off (time between snapshots, 1 ms). B, Post-slip vibration for the example in (A). C, Best fit and 0.95 confidence intervals for 8 whisker slips.

Whiskers vibrate when force is applied to them. These vibrations decay based on damping properties of the whisker and the follicle. To measure the damping coefficient *α* and first eigenfrequency *ω* of the C2 whisker, we quantified the decay of whisker curvature change following rare events where the whisker slipped past the pole. The pole was 10 mm from the follicle base and curvature measured 6 mm from follicle base (Fig 3a). These slips provide a large, sharp impulse to the whisker after which the whisker vibrates freely. We describe these vibrations using the oscillatory damped function: $f\left(t\right)=A\;\text{sin}\;\left(\sqrt{\omega^{2}-{\left(\alpha /2\right)}^{2}}t+\phi \right)\;\text{exp}\left(-\alpha t/2\right)$, where *A* is the amplitude of the oscillations and *ϕ* is their phase at *t* = 0. Eight slip-offs were fit, each giving an estimate of the oscillation frequency and damping of the whisker (Fig 3b and 3c). Damping and eigenfrequencies estimates were independent of force magnitude, the point of force application and the point of whisker curvature measurement. The mean damping value, fitted to the decay of the exponent was *α* = 430 ± 120 rad/s and the mean angular frequency of vibration was *ω* = 962 ± 50 rad/s (153 ± 8 Hz). This is well within the range with which mechanosensory neurons in the follicle can fire in every cycle [27]. Since Eq (24) relates the vibration (angular) frequency to Young’s modulus *E*, using the measured whisker’s parameters, length *L* = 17.14 mm, radius at base *R* = 37.15 *μ*m, and density *ρ* = 1.0g/cm^3^ we can estimate *E* for this whisker to be 3.04 GPa. This number is near the center of the range of values reported for rat whiskers 3.34 ± 1.48 GPa [28].

In the analytical modeling examples throughout the remainder of this paper, we selected parameters close to those extracted from these measurements. All whiskers were linearly tapered cones truncated at 0.95 of the extrapolated length (*i*.*e*., *ℓ*/*L* = 0.05) unless otherwise specified. We set length to be *L* = 18mm, base radius *R* = 37 *μ*m, density *ρ* = 1.0 g/cm^3^, damping *α* = 430 rad/s, and Young’s modulus *E* = 3.00 GPa.

### Analytical solution for whisker vibrations from touch

**Fig 4:**
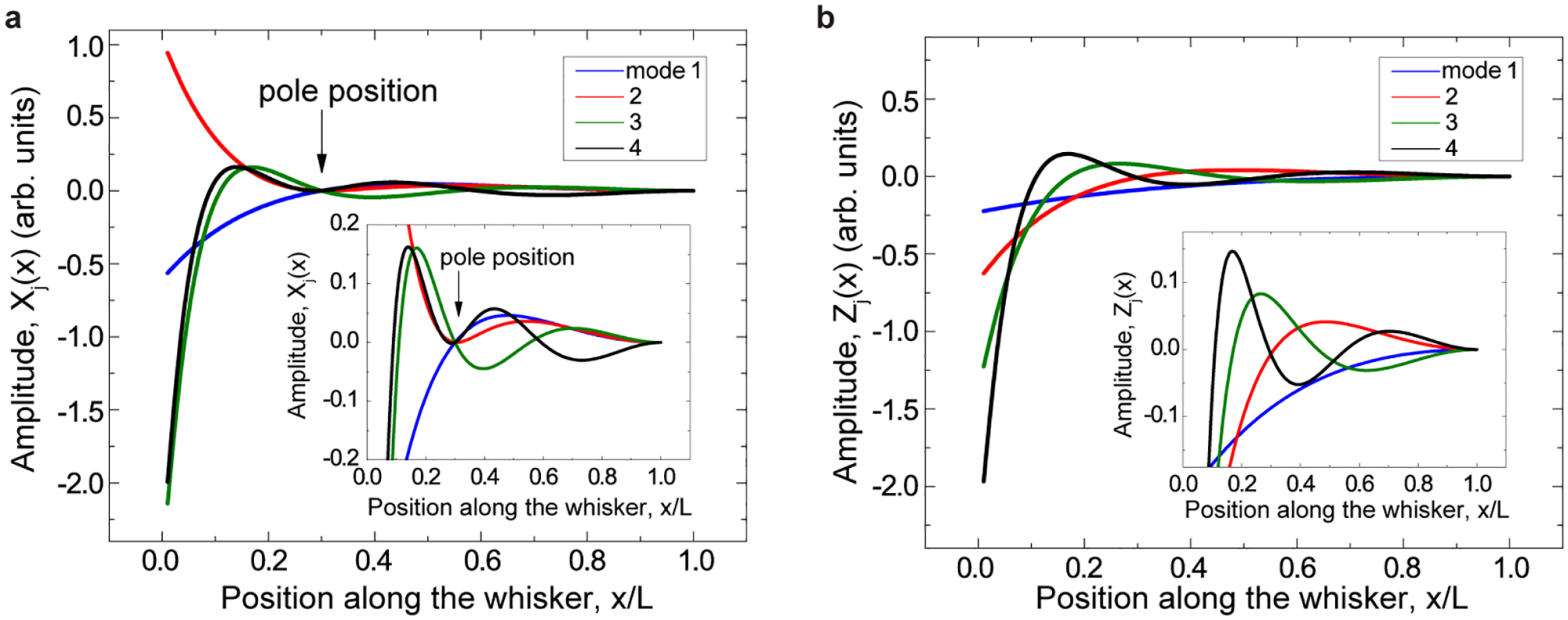
Four lowest eigenmodes for a full-length conical whisker with fixed-base free-tip boundary conditions. A, Pole located at *c*/*L* = 0.3, as indicated by arrow. B, Free vibration of the whisker in absence of the pole. Insets: The same four eigenmodes magnified for clarity.

To understand how vibrational dynamics of whiskers could influence tactile perception, we first calculated the eigenmodes and eigenfrequencies of a model whisker (*i*.*e*. a linearly tapered cone) during two sets of boundary conditions, when the whisker is in contact with a thin cylindrical pole (Fig 4a), or where it is vibrating freely (Fig 4b). In both cases, the follicle is fixed. During touch, all eigenmodes share a node at the follicle base and at the point of contact.

**Fig 5:**
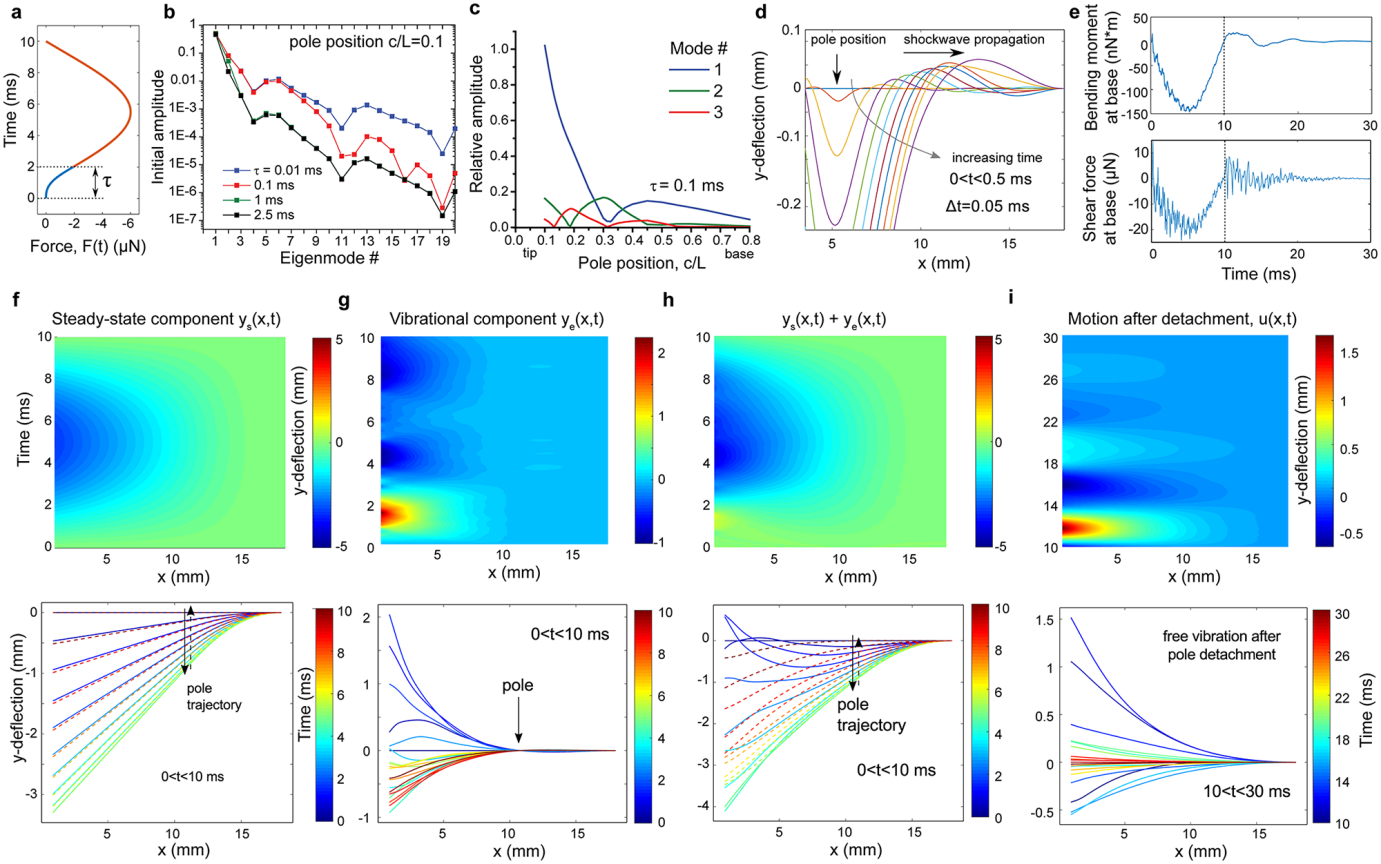
Model response to touch. A. Time dependent profile of the applied force modeled by a truncated Gaussian (Eq (77)) and a smooth connecting function *F*_*s*_(*t*) (Eq (79)) that models the touching event at early times, 0 ≤ *t* ≤ *τ*. For clarity, *τ* is exaggerated in the figure. B. Amplitudes of the eigenmodes excited by pole impact as a function of the eigenmode number calculated for different *τ*. C. The relative excitation of eigenmodes is dependent on location of contact along the whisker. D. Touch onset propagates a vibrational wave towards the base of the whisker. E. Bending moment and shear force at the follicle during, 0 ≤ *t* ≤ 10 ms, and after, *t* > 10 ms, touch calculated for pole position *c*/*L* = 0.6. F-I. Time-dependent displacement of a 5% trimmed whisker during and after typical interactions with pole located at *c*/*L* = 0.6. F. Top: steady state component, *y*_*s*_(*x*, *t*), of the whisker displacement during contact with pole, 0 ≤ *t* ≤ *t*_*f*_, *t*_*f*_ = 10 ms. Bottom: solid and dashed lines show *y*_*s*_(*x*, *t*) sampled with 0.5 ms steps during the forward and backward motion, respectively. G. Top: vibrational component of the whisker displacement, *y*_*e*_(*x*, *t*), during contact with pole. Bottom: vibrational component *y*_*e*_(*x*, *t*) sampled with 0.5 ms steps. H. Top: sum of the steady-state and vibrational components. Bottom: solid and dashed lines show the total whisker shape sampled with 0.5 ms steps during the increase and decrease of the force, respectively. The time colorbar shown in F applies to all bottom panels F-I. I. Top: free whisker motion after the pole detachment, *t* > *t*_*f*_. Bottom: whisker shape for the same time interval sampled with 0.5 ms time steps. The value *τ* = 0.1 ms was used in panels C-I.

We next developed analytical solutions for the vibration of a mouse whisker during object contact at a single point along the whisker’s length, and for free vibration (see Materials and Methods for a complete description). We modeled the timecourse of touch forces based on typical observed touches (Figs 2d and 5a), with the shape of a truncated Gaussian curve. We modeled touch onset as a smooth connecting function with one free parameter, *τ*, the duration of time between zero force and the main Gaussian (Fig 5a). This is in contrast to previous work [24], which treat impact as an instantaneous event. Excitation of eigenmodes is sensitive to the duration of the transition period, whereas dynamics at moment of impact are beyond our temporal resolution of observation. Therefore, we performed a parameter sensitivity analysis of *τ* on eigenmode excitation. The magnitude of excitation of the first six eigenmodes was insensitive to shortening *τ* below 0.1 ms (Fig 5b). Eigenmodes with order above six are >100x weaker than primary and secondary modes, and have higher frequency than what could plausibly be sensed by mechanoreceptors in the follicle. Therefore, we defined *τ* to be 0.1 ms for all later analysis.

Excitation of eigenmodes is also sensitive to the location of contact, with the fundamental mode dominating higher modes for most contact positions, except around *c*/*L* = 0.3, where the second mode excitation becomes most prominent (Fig 5c). The relative amplitude of these modes affects the temporal pattern of peak vibrational forces at the follicle.

A prominent feature of observed touches and prior models of whisker vibrations is the propagation of a wave from the point of contact towards follicle immediately after the onset of touch [24]. Our model recapitulates this phenomenon (Fig 5d). Shear force and bending moment at the follicle drive mechanotransduction [22]. Our model describes the vibrational components of shear force and bending moment (Fig 5e) at the follicle following contact onset and offset. These components likely drive mechanotransduction. Contact with a pole causes bending and deflection from applied steady-state force and vibration. Our model provides a complete solution for the displacement of the whisker along its entire length, including beyond the object contact point, decomposed to the steady-state component (Fig 5f), the vibrational component (Fig 5g), and sum of these during (Fig 5h) and after contact (Fig 5i).

**Fig 6:**
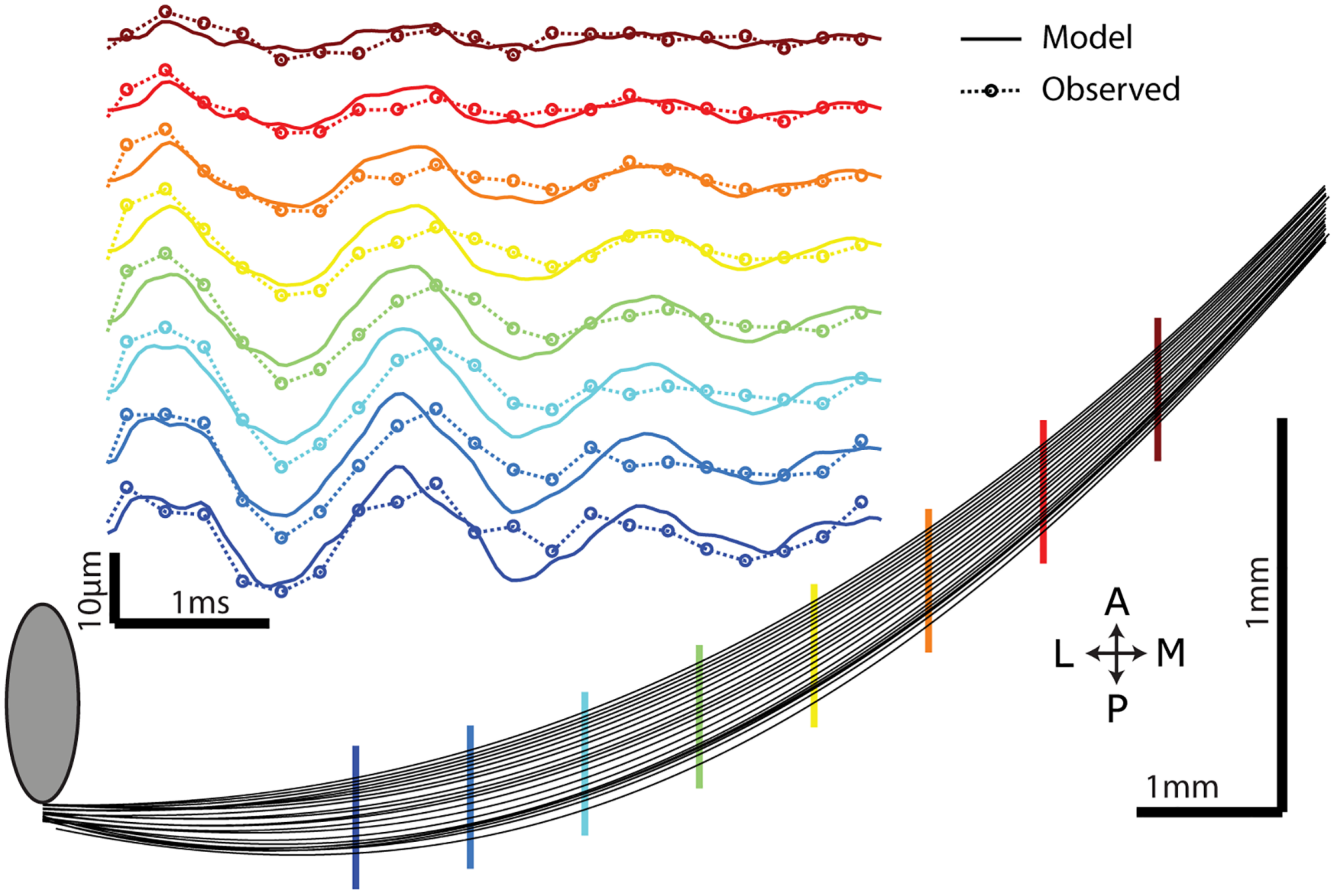
Comparing model and observation. Traced high speed imaging (4000 fps) of whisker displacement and deformation during touch. Each line is a whisker position during the first 5 ms from onset (0.25ms timesteps). Y-axis is expanded. This whisker had an intrinsic curvature that bent towards the anterior of the head, in the direction of protraction. Compass indicates anteroposterior and mediolateral axes. Inset, zero free parameter model predictions and observed vibrational deformation through time at eight color coded locations along the whisker during this example touch.

To validate the accuracy of our model, we performed 4000 frame per second imaging of active whisker touch during object localization. Following the experiment, we measured the whisker dimensions *L* = 15.726mm, radius at base *R* = 38.96 microns. Using the whisker properties, the location of the pole (*c*/*L* = 0.356) and the velocity of whisker at the contact point (86mm/s) we calculated the vibrational response of the whisker with zero free parameters. Remarkably, observed responses had appropriate sign, shape, phase and amplitude (Fig 6).

**Fig 7:**
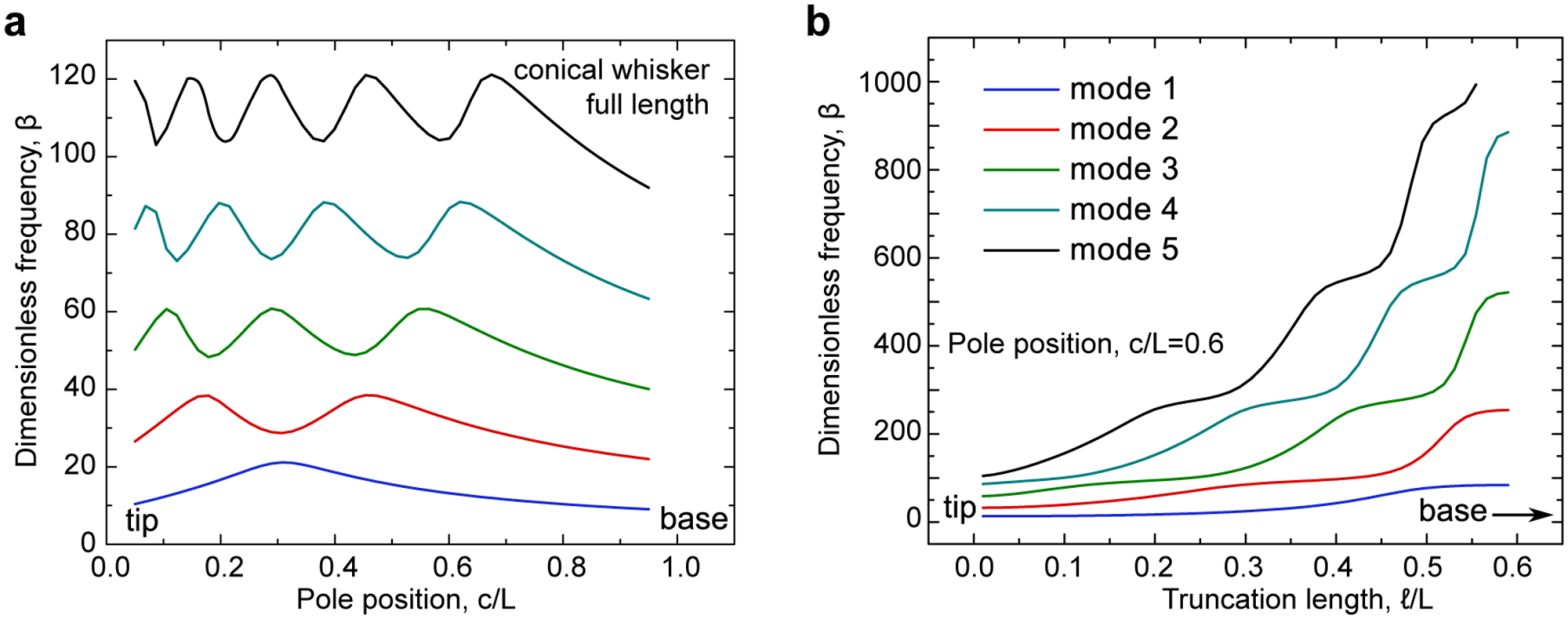
Dependencies of eigenfrequencies. A, The dependence of the five lowest dimensionless eigenfrequencies, *β*_*j*_, on pole position for a full-length conical whisker. B, The dependence of the five lowest dimensionless eigenfrequencies on the truncation length for a fixed pole position *c*/*L* = 0.6.

For vibration to serve as a cue for object distance during contact with a single whisker, there must be some detectable distance-dependent change in whisker vibration. This could manifest as a change in vibration frequency or magnitude. To determine if such a cue could be present, we calculated the frequencies of the first five eigenmodes during object contact. We found a strong dependence of vibration frequency on the distance between contact point and follicle, with the first mode increasing in frequency by 2.31x between contact near the base (*c*/*L* = 0.95) and *c*/*L* = 0.32 (Fig 7a). This is qualitatively similar to results for a cylindrical whisker [13], albeit with a less pronounced change in frequency. Here, since the vibration is of a beam with free tip, the distance-frequency relationship is non-monotonic, increasing away from base then decreasing as the contact point approaches the tip. Higher eigenmodes show multi-peaked relationships, with number of peaks equal to the order of the mode, and less relative modulation with increasing order. In a frictionless system, this relationship is independent of touch force or *τ*, making it robust to variation in how firmly or quickly the mouse strikes the pole. Truncation of the whisker also affected the eigenmode frequency, with modest effects on the first mode, and increasingly strong effects on higher modes (Fig 7b).

**Fig 8:**
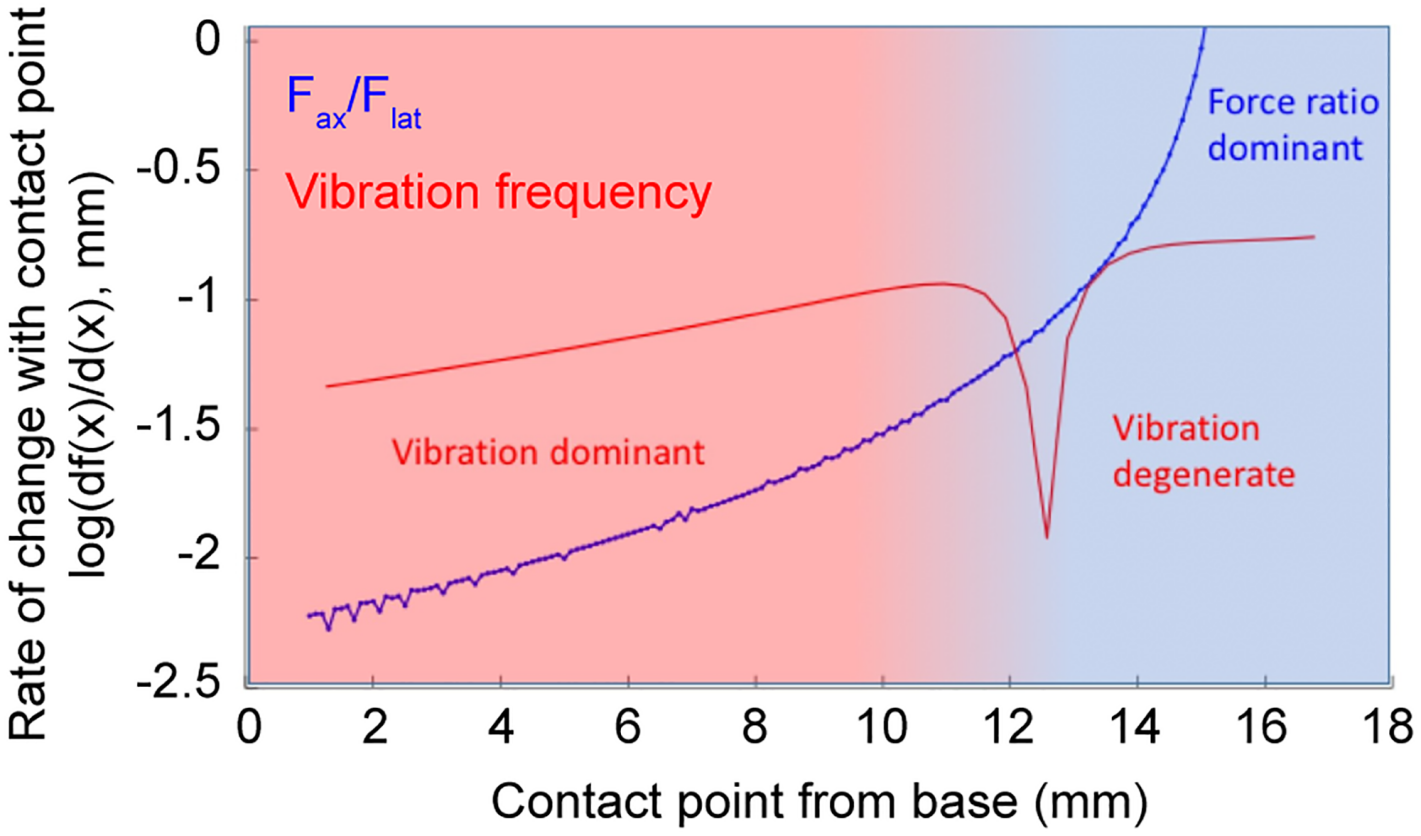
Comparison of distance dependence of vibration frequency and force component ratios. Red, the rate of change of fundamental vibrational frequency against contact distance along the whisker length. Blue, the rate of change of force ratios against contact distance along the whisker length. Log base 10 y-axis. There is little change in axial-to-lateral force, *F*_ax_/*F*_lat_, (< 1% / mm) in proximal contact positions due to whisker stiffness preventing substantial curvature during touch. However, the fundamental frequency of vibration changes substantially (5 – 10% / mm) with contact position for a large range of contacts out to *c*/*L* = 0.3. At more distal contact positions, the first eigenfrequency falls and is degenerate with proximal frequencies. The rate of change of force component ratios accelerates towards the tip.

Could mice determine the distance to the pole using vibration frequency rather than the ratio of axial to lateral forces (*i*.*e*. the angle of the touch force [18])? To assess this, we compared the relative rate of change in vibration frequency and force ratios as the contact point moves along the whisker (Fig 8). In the proximal half of the whisker, fundamental vibration frequency changes 7.4–4.1x more quickly with increasing distance (4.5–10% / mm) than force ratios (0.6–2.2% / mm). Near *c*/*L* = 0.3, vibrational frequency becomes more steady, before changing more rapidly again towards the whisker tip. The fundamental frequency near the tip is degenerate with frequency in the proximal 2/3 of the whisker, though including higher order modes could uniquely resolve object distance. In contrast, the rate of change in force ratios increases rapidly for contacts beyond 2/3 of whisker length, exceeding 100% / mm near the tip. This is consistent with the distal third of the whisker being several orders of magnitude more flexible than the proximal third. Overall, this suggests that vibration frequency would be a more salient cue for object distance for contacts in the proximal half of the whisker than axial to lateral force ratios, provided similar detection sensitivity.

**Fig 9:**
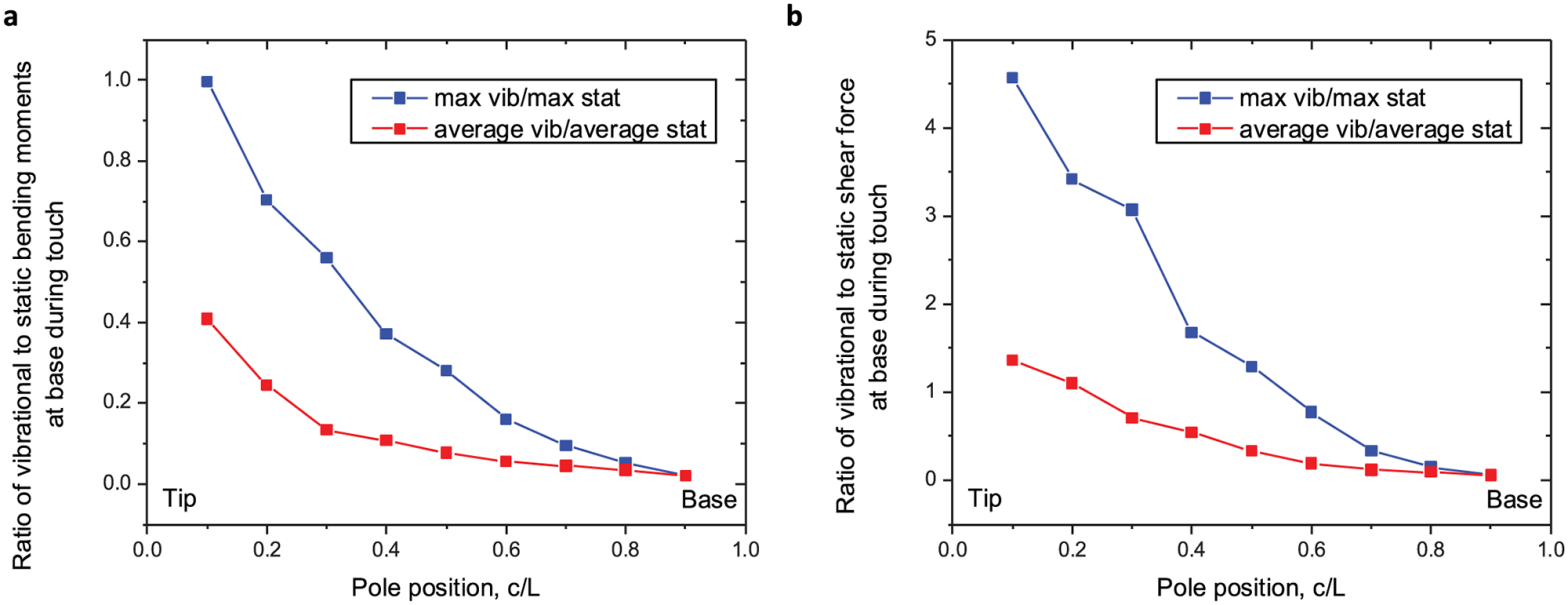
A. Touches evoke quasi-static and vibrational bending moments at the base. The ratio of quasi-static and dynamic bending moments increases dramatically toward the tip. B. Similarly, the ratio of quasi-static and dynamic shear forces increases dramatically toward the tip.

Vibration magnitude could also serve as a contact distance cue. Touches with equal push angle generate dramatically reduced steady-state forces with increasing contact distance [16, 25]. The magnitude of vibration forces are less dependent on contact distance. We illustrate this by calculating the ratio of vibration to steady state shear forces and bending moments for contacts at varying distance from the whisker base. These ratios increase dramatically as the contact point moves from base to tip (Fig 9). Both the average and maximum vibrational bending moment and shear force are a small fraction the steady-state component for very close contacts (*c*/*L* = 0.9; shear 0.057 average, 0.057 maximum; bending moment 0.020 average, 0.021 maximum). Near the tip, these forces can approach or exceed the steady state component (*c*/*L* = 0.1; shear 1.36 average, 4.57 maximum; bending moment 0.409 average, 0.996 maximum). Since mice have distinct sensing afferents for fast vs. slowly changing touch forces [20, 21], comparison of activity between these afferents is a plausible additional mechanism for determining object distance.

## Discussion

### Accuracy of the mechanical model

Our model of whiskers deformed by time-varying point forces relies on several simplifying assumptions. First, we used the Euler-Bernoulli beam theory for thin beams, which ignores shear terms. Rodent whiskers have a slenderness ratio of >> 100, a regime where shear terms are irrelevant [29].

We assume a constant path length from follicle to point of force application. The majority of touches have a change in push angle of < 15 degrees, and the peak increase in path length during touch was < 1% for 81% of touches (Fig 2c), justifying our use of the small-angle approximation [30]. For the 3% of touches with peak angle difference of > 50 degrees, a more sophisticated non-linear model may be necessary for accurate calculation of vibration.

We treat the whisker in two dimensions. We also ignore friction, since the applied forces are assumed to be normal to the longitudinal axis of the whisker [16]. Frictional forces could tension the whisker, altering vibrational frequencies. Full treatment of vibrations in three dimensions, including torsional movement [31], intrinsic whisker curvature [32], and friction would require a numerical approach [33].

We use approximate boundary conditions at the follicle. The follicle holding the whisker has unknown and potentially variable compliance during whisking [34–37]. The angle of the whisker relative to the follicle is thus fixed, whereas bending moment (directly proportional to the whisker curvature and the magnitude of the applied force), and shear force (the rate of change of the curvature) vary. Since the parameters relevant to object localization [18], including bending moment, can be calculated for the fixed end boundary condition, we focused our analysis on this case.

Mouse whiskers are not quite linear cones, but are thinner in the middle than a linear fit [14]. This has an appreciable impact on the bending angle, due to the fourth power dependence of stiffness on radius, but the effect on the vibrational modes is estimated to be relatively small.

We assumed Young’s modulus to be constant along the whisker. Rat and mouse whiskers have an inner hollow medulla, filled with spongy material. This could cause a difference in effective Young’s Modulus between proximal and distal halves of the whisker [28]. The estimates of the damping coefficient, vibration frequency and steady state displacement all depend on Young’s Modulus.

Our reference frame fixes the x-axis along the long axis of the whisker. From this perspective, the deflections produced by a whisker rotated into a pole (as in active touch) are equivalent to a pole moving into a whisker held steady (as in passive deflection). Differences in blood sinus pressure and muscle tension between passive and active touch could potentially influence follicle compliance, damping, and how forces applied to the follicle are transduced by mechanoreceptors and perceived by the brain [21, 38]. Since all touches examined in this project were by produced by head-fixed mice using whisking to locate objects, our results estimating follicle compliance and damping are best suited for modeling vibrations of active touch. Accurate calculation of vibrations from passive touch may require different damping coefficients.

There are also several sources of potential experimental measurement error. Due to droop and whisker elevation change, the single top-down projection in our dataset results in an underestimate of the true path length to contact, *c*/*L*, in the behavioral measurements (Fig 2). Dual view imaging shows that this error is <4 percent for touches in the proximal half of the whisker, but increases beyond that point. The fur on the face obscures the whisker insertion to the follicle. We compensated for these factors by assuming the whisker continued into the fur an additional 1–1.75mm depending on fur length, justified by our prior high-resolution measurements of mouse whisker shape [14].

Whisker displacement following touch is composed of quasi-steady state and vibration parts. Mice actively control the quasi-steady state trajectory in an irregular manner, so we must estimate this part by smooth fits of the trajectory across time. Our choice of fit influences the residual vibration component, particularly at the edges of the measured period.

Damping of whisker vibrations (Figs 3 and 6) could arise from dissipation within the whisker, the follicle, the whisker-object interface, friction and coupling to tissue surrounding the follicle [24, 30]. We modeled damping during free vibration using a single parameter linearly applied to each vibrational mode. Changes in path length *c* during strong or distal contacts could cause destructive interference of the range of eigenmodes excited during a single contact and frictional dissipation, reducing vibration.

Given these caveats, our model predicts the phase, absolute and relative amplitude of vibration by active whisker touch well (Fig 6). There is a small mismatch in frequency and damping, which we expect are due to estimating damping during free vibration, simplified whisker geometry, curve fitting of whisker traces to overcome sub-pixel alignment noise, and uncompensated active accelerations of the whisker by the mouse. The accuracy of model is particularly impressive because it is untuned, with zero free parameters.

### Comparison to other work

Our model diverges from other recent work [20, 24] in several ways. We model the whisker in its entirety, including beyond the contact point, allowing placement of the pole at any location along the whisker. This is especially important for studying vibrational cues in close proximity to the whisker base. We consider a Gaussian form of the force time-dependence mimicking the experiment, but any force profile can be treated with our methods. We use a frequency-independent damping constant, which is reasonable since the higher vibrational eigenfrequencies are outside the animal’s perception limits. Critically, we avoid singularity in the whisker acceleration upon impact by using a smooth force onset that keeps the first two time-derivatives of the force continuous.

Most importantly, we analytically solve the spatial eigenmodes of conical whiskers, with and without truncation in both motion phases, in contact with pole and in free motion. The analytical solution provides closed-form expressions of the eigenmodes, which is significantly more robust and less computationally expensive than obtaining them numerically. Using the closed-form expressions avoids numerical errors, which becomes increasingly important for higher eigenmodes needed for the expansion of vibrational motion trajectories. Numerical instabilities limited the number of modes considered in prior work [20]. Thus, our approach allows a more complete view of the time-evolution of whisker vibration. The derived analytical solutions can be easily reproduced and used by others within Mathematica or MATLAB package without additional programming. The resultant expressions are transparent, can be easily manipulated, and subjected to various boundary conditions. As a result, the developed analytical approach can be easily generalized and adjusted for more complex experimental conditions, such as multi-point touching, slipping, or sliding.

### Contributions of transverse vibrations to whisker-dependent behaviors

Identifying the location of objects relative to the face is important for social interaction, foraging, navigation, and other behaviors. Rodents actively sweep their whiskers forward and back during these behaviors. In head-centered spherical coordinates, the radial distance, azimuthal angle, and polar angle of objects can determined by distinct active touch strategies. Polar angle could be determined by labelled-line activation of whiskers across different rows [39], which extend to different elevations above and below the face. Azimuthal angle could be determined by the number of touches in a whisking bout [40], the timing of touch relative to neuronal phase-locked loops [41], the roll angle of the whisker at touch [31, 42], or an integration of whisking amplitude and midpoint signals with phase amplified touch responses [43]. Radial distance could be determined with low resolution by labelled line activation of whiskers of different lengths [44]. Higher resolution distance discrimination requires other cues, potentially including vibration.

Quasi-static analysis has shown that axial forces pushing the whisker into the face could contribute to tactile perception [17, 18, 45]. However, axial vibrational displacements and forces are expected to be negligible. Axial frequencies of whiskers are much higher than transverse frequencies. The difference is controlled by the factor *r*/*L* where *r* is the whisker radius and *L* is its length (compare Timoshenko Eq. 5.4 on page 36 and Eq. 5.102 on page 421). This gives a factor 160 for our whiskers, corresponding to vibration frequencies >100 kHz. Mechanoreceptors are not sensitive to frequencies in this band. In addition, the associated displacements are four orders of magnitude smaller compared to transverse vibrations, because the displacement is inversely proportional to the square of eigenmode frequency (Timoshenko Eq. c on page 73). Thus, radial displacements from vibration are expected to be on the order of one hundred nanometers. While steady state axial forces likely play important roles in tactile sensation, axial vibrations can be neglected as tactile cues.

Mice can determine radial distance with a single touch of an individual whisker [18, 19]. Experimental manipulation of the compliance of the object and stiffness of the whisker supports a decision model where the ratio of quasi-static axial and lateral forces are used for discrimination [18]. Could vibrational cues also contribute to distance discrimination? At least two potential strategies could be used.

The first relies on comparing the temporal patterns of spikes evoked by touch (Fig 7). The timing precision of evoked spikes is sufficiently high within primary sensory neurons (median spike jitter 17.4 *μ*s, [27]) to encode vibration frequency and pattern changes from the sum of the first few eigenmodes. High temporal fidelity is a hallmark of intermediate locations between follicle and cortex [46–48]. Vibrational frequency can be used to discriminate contact distance in isolated preparations [5, 20]. Ideal observers of spike trains can use precise timing in primary afferents to decode stimulus features [22, 49–51]. Changes in synchrony across multiple afferents could potentially be read out by downstream neurons in S1 or other brain regions to produce a neural representation of vibration frequency and corresponding object distance. Since we do not know the relative sensitivity mice have for pitch shift vs. force ratio changes on whiskers, it remains unclear whether the animal has the capability of using sub millisecond differences in tactile sensory input patterns to influence perception and drive behavior.

The second strategy involves comparison of the amplitude of two streams of sensory input, static forces from bending, and dynamic forces from vibration. As mentioned above, these are likely selectively encoded by slowly and rapidly adapting mechanosensory afferents. Due to the increased flexibility of whiskers towards the tip, steady-state forces during contact are two or more orders of magnitude smaller during contacts near the tip than near the base. In contrast, the magnitude of vibrational forces are primarily dependent on whisker velocity at contact, which is less distance dependent. Thus, the relative magnitude of vibration to steady state forces could provide another important cue to distinguish radial distance (Fig 8).

Since the mammalian brain exhibits flexible learning, it seems likely that which cues drive behavior will be a combination of static and dynamic signals that reflect task demands, motivation, and training history. Behavioral experiments could be used to ascertain if and how mice use vibrations to judge radial object distance. Vibration, but not steady state force, depends on whisker properties beyond the point of contact (Fig 6). Thus, whisker trimming will increase vibration frequency without affecting steady state forces. If frequency serves as a critical cue, our results predict that in mice trained to discriminate radial distances in the proximal half of a single whisker, reported object distance will increase if the whisker tip is trimmed off, quantifiable by a shift in psychometric curves.

Determining if and how neural circuits compare vibration and quasi-static forces to influence perception of object distance has recently become a tractable problem. Recent work has measured firing properties of identified classes of mechanosensory afferents from the follicle during whisker motion [22]. Genetic labelling has identified a diversity of convergence of slowly and rapidly adapting afferents onto second-order projection neurons from mouse whisker follicles [52]. Given appropriate upstream connectivity, these could provide an appropriate set of sensory representations to make comparisons between dynamic and static information during active touch. Further dissection of these circuits should be possible with advanced genetic methods. Thus, our results computing whisker vibrations based on real whisker touches during active sensing provides a strong foundation and justification for future investigations into how neural circuits perform computations on tactile sensory input to produce perception.

## Materials and methods

### Ethics statement

All procedures were in accordance with protocols approved by the Institutional Animal Care and Use Committees of the University of Southern California (Protocol 20169) and Janelia Research Campus (Protocol 11–71).

### Mathematical notation and coordinate system

1. The *x*-axis is the line between whisker tip and whisker base when the whisker is at rest (Fig 1b).
2. *ℓ* is the location of the truncated whisker tip, or the truncation length (the extrapolated virtual tip is located at the origin).
3. *L* is the distance from the origin to the whisker base.
4. *c* is the point of force application (pole position).
5. *R* is the whisker base radius at *x* = *L*.
6. *r*(*x*) = *ξx* is the whisker radius at point *x*, where *ξ* = *R*/*L*.
7. *E* is Young’s modulus of the whisker.
8. *ρ* is the volume density of the whisker.
9. *z*, *γ*, *β*, *η* = *qx*, *qℓ*, *qL*, *qc* are four of the previous variables written in a dimensionless form, where *q* = (4*ρω*^2^/*Eξ*^2^)^1/2^ and *ω* is the eigenmode angular frequency.
10. *M*(*x*, *t*) is the bending moment at position *x* and time *t* created by a force applied at *c*.
11. *I*(*x*) = (*π*/4)*r*^4^(*x*) is the area moment of inertia at point *x*. At the whisker base, *I*(*L*) = (*π*/4)*R*^4^ ≡ *I*_0_.
12. *μ*(*x*) = *ρπr*^2^(*x*) = *ρπξ*^2^ *x*^2^ is the linear density of the whisker.
13. *α* is the frequency-independent damping constant.
14. In the small angle approximation, the applied force *F* ≈ *F*_⊥_, the force component perpendicular to the whisker axis.
15. *f*(*x*, *t*) is the linear density of the force applied by pole.
16. *t*_*f*_ is the duration of contact with pole.
17. *τ* is the duration of the smooth force onset, *τ* ≪ *t*_*f*_.
18. *y*(*x*, *y*) is the total whisker displacement from straight line during contact with pole, with *y*_*s*_(*x*, *t*) and *y*_*e*_(*x*, *t*) being the steady-state and vibrational components of *y*(*x*, *t*), respectively.
19. *u*(*x*, *y*) is the total whisker displacement during the free motion following detachment from pole.
20. *X*_*j*_(*x*) and *ω*_*j*_ are the spatial function and angular frequency of the *j*-th vibrational eigenmode of a fixed-base free-tip conical whisker with a simple support at the point of force application, *x* = *c*.
21. *Z*_*j*_(*x*) and $\varpi_{j}$ are the spatial function and angular frequency of the *j*-th eigenmode of a freely vibrating fixed-base free-tip conical whisker.
22. *ϕ*_*j*_(*t*) and *φ*_*j*_(*t*) are time-dependent coefficients in expansion of the whisker motion in spatial eigenmodes during forced and free motion, respectively.

### Derivation of the analytic solution for whisker dynamics

#### Static bending of a conical whisker in the small angle approximation

We start the derivation from a description of the static whisker bending under applied force. In the small angle approximation, the shape of a thin rod (*i*.*e*., a whisker) in response to an applied force is given by:

$$\begin{array}{c} \frac{d^{2}y_{s}}{dx^{2}}=\frac{M(x)}{EI(x)}\;, \end{array}$$ (1)

where *y*_*s*_ is the steady state displacement of the whisker from the *x* axis (the straight line that connects the whisker tip and base in the absence of force; see Fig 1), and *E* is the Young’s modulus. The area moment of inertia of a conical whisker is *I*(*x*) = (*π*/4)*r*^4^(*x*), where *r*(*x*) = *ξx* is the whisker radius at position *x*, with *ξ* being a constant. We place the whisker base at *x* = *L* and its trimmed tip at *x* = *ℓ*, such that the extrapolated virtual tip is at *x* = 0. For a full-length whisker, *ℓ* = 0 and the real whisker tip is at the origin. The force *F* is applied at point *x* = *c*, and we take it positive when it’s applied in the positive *y*-direction (upwards). The bending moment created by the applied force can be expressed as:

$$\begin{array}{c} M\left(x\right)=\{\begin{array}{cc} 0 & \ell \leq x\leq c\\ (x-c)F & c\leq x\leq L \end{array}\;. \end{array}$$ (2)

Integrating the differential Eq (1) twice results in:

$$\begin{array}{c} \begin{array}{c} y_{s}\left(x\right)=\frac{4F}{\pi E\xi^{4}}(\frac{1}{2x}-\frac{c}{6x^{2}}+C_{1}x+C_{2})\;c\leq x\leq L\;,\\ y_{s}^{\prime }\left(x\right)=\frac{4F}{\pi E\xi^{4}}(-\frac{1}{2x^{2}}+\frac{c}{3x^{3}}+C_{1})\;c\leq x\leq L\;. \end{array} \end{array}$$ (3)

In our calculations we assume fixed-base boundary conditions:

$$\begin{array}{c} y\left(L\right)=0,\;y^{\prime }\left(L\right)=0\;, \end{array}$$ (4)

which allow us to determine the integration constants *C*_1_ and *C*_2_. This leads to the following expressions for *y*_*s*_(*x*) and $y_{s}^{\prime }\left(x\right)$ in the interval *c* ≤ *x* ≤ *L*:

$$\begin{array}{c} \begin{array}{c} y_{s}\left(x\right)=\frac{2F}{3\pi E\xi^{4}}(\frac{3x-c}{x^{2}}+\frac{\left(3L-2c)x+3L(c-2L\right)}{L^{3}}),\\ y_{s}^{\prime }\left(x\right)=\frac{2F}{3\pi E\xi^{4}}(\frac{2c-3x}{x^{3}}+\frac{3L-2c}{L^{3}})\;. \end{array} \end{array}$$ (5)

At the point of force application, *x* = *c*, we calculate *y*_*s*_(*c*) and $y_{s}^{\prime }\left(c\right)$:

$$\begin{array}{c} y_{s}\left(c\right)=\frac{4F}{3\pi E\xi^{4}L^{3}c}{\left(L-c\right)}^{3}\;,\\ y_{s}^{\prime }\left(c\right)=\frac{2F}{3\pi E\xi^{4}L^{3}c^{2}}\left(3Lc^{2}-L^{3}-2c^{3}\right)\;. \end{array}$$ (6)

which allows us to define *y*_*s*_(*x*) in the interval *ℓ* ≤ *x* ≤ *c* as:

$$\begin{array}{c} y_{s}\left(x\right)=y_{s}^{\prime }\left(c\right)\left(x-c\right)+y_{s}\left(c\right)\;. \end{array}$$ (7)

#### Eigenmodes and eigenfrequencies of a conical whisker

In this section we derive analytical expressions for eigenmodes and eigenfrequencies of truncated and full-length conical whiskers, with and without contact with pole. The whisker is modeled as an elastic conical beam and the pole is modeled by a simple support placed at the point of force application.

#### General solution

The vibrational eigenmodes of a conical whisker are determined by the following differential equation [53]:

$$\begin{array}{c} \frac{\partial^{2}}{\partial x^{2}}(EI\left(x\right)\frac{\partial^{2}y}{\partial x^{2}})=-\mu \left(x\right)\frac{\partial^{2}y}{\partial t^{2}}\;, \end{array}$$ (8)

which is the homogeneous part of Eq (55) below with force density and damping constant set to zero, *f*(*x*, *t*) ≡ 0 and *α* = 0, respectively. Here, *y* = *y*(*x*, *t*) is the whisker displacement from the *x*-axis and *μ*(*x*) is the whisker’s linear density. For conical geometry we have:

$$\begin{array}{c} \mu \left(x\right)=\rho \pi \xi^{2}x^{2},\;I\left(x\right)=\left(\pi /4\right)\xi^{4}x^{4}\;. \end{array}$$ (9)

Substituting Eq (9) in Eq (8) we obtain:

$$\begin{array}{c} \frac{\partial^{2}}{\partial x^{2}}(x^{4}\frac{\partial^{2}y}{\partial x^{2}})=-\frac{4\rho }{E\xi^{2}}x^{2}\frac{\partial^{2}y}{\partial t^{2}}\;. \end{array}$$ (10)

Separating the spatial and temporal dependences in the solution as *y*(*x*, *t*) = *X*(*x*)*g*(*t*), with *g*(*t*) having harmonic time dependence satisfying $\ddot{g}\left(t\right)=-\omega^{2}g\left(t\right)$, we obtain the equation for the spatial part alone:

$$\begin{array}{c} \frac{\partial^{2}}{\partial x^{2}}(x^{4}\frac{\partial^{2}X}{\partial x^{2}})=\frac{4\rho \omega^{2}}{E\xi^{2}}x^{2}X\left(x\right)\;. \end{array}$$ (11)

Introducing a new dimensionless variable:

$$\begin{array}{c} z=qx\;\text{with}\;q^{2}=\frac{4\rho \omega^{2}}{E\xi^{2}}, \end{array}$$ (12)

and a new function:

$$\begin{array}{c} \chi (z)=X(x)=X(z/q), \end{array}$$ (13)

we transform Eq (11) into a tidy dimensionless form:

$$\begin{array}{c} \frac{\partial^{2}}{\partial z^{2}}(z^{4}\frac{\partial^{2}\chi }{\partial z^{2}})=z^{2}\chi \left(z\right)\;. \end{array}$$ (14)

It has been demonstrated [54] that the general solution of the fourth-order Eq (14) can be constructed from two linearly-independent solutions of the Bessel equation of order two:

$$\begin{array}{c} x^{2}\frac{d^{2}y}{dx^{2}}+x\frac{dy}{dx}+\left(x^{2}-2^{2}\right)y=0\;, \end{array}$$ (15)

and from two linearly-independent solutions of the modified Bessel equation of order two:

$$\begin{array}{c} x^{2}\frac{d^{2}y}{dx^{2}}+x\frac{dy}{dx}-\left(x^{2}+2^{2}\right)y=0\;. \end{array}$$ (16)

The two independent solutions of Eq (15) are the Bessel functions of the first and second kind, *J*_2_ and *Y*_2_. The two solutions of Eq (16) are the modified Bessel functions of the first and second kind, *I*_2_ and *K*_2_. The four linearly-independent solutions of Eq (14) have been shown [54] to be of the form:

$$\begin{array}{c} \frac{J_{2}\left(2\sqrt{z}\right)}{z},\;\frac{Y_{2}\left(2\sqrt{z}\right)}{z},\;\frac{I_{2}\left(2\sqrt{z}\right)}{z},\;\frac{K_{2}\left(2\sqrt{z}\right)}{z}\;, \end{array}$$ (17)

which can be directly checked by their substitution into Eq (14).

#### Freely vibrating full-length conical whisker

Let us start from considering the eigenmodes of a freely vibrating full-length (not truncated) conical whisker. The general solution of Eq (14) can be written as:

$$\begin{array}{c} \chi \left(z\right)=c_{1}\frac{J_{2}\left(2\sqrt{z}\right)}{z}+c_{2}\frac{Y_{2}\left(2\sqrt{z}\right)}{z}+c_{3}\frac{I_{2}\left(2\sqrt{z}\right)}{z}+c_{4}\frac{K_{2}\left(2\sqrt{z}\right)}{z}\;. \end{array}$$ (18)

Using the Bessel function recurrence relations [55], one can write the first three derivatives of the general solution:

$$\begin{array}{c} \begin{array}{c} \chi^{\prime }\left(z\right)=-c_{1}\frac{J_{3}\left(2\sqrt{z}\right)}{z^{3/2}}-c_{2}\frac{Y_{3}\left(2\sqrt{z}\right)}{z^{3/2}}+c_{3}\frac{I_{3}\left(2\sqrt{z}\right)}{z^{3/2}}-c_{4}\frac{K_{3}\left(2\sqrt{z}\right)}{z^{3/2}}\;,\\ \chi^{\prime \prime }\left(z\right)=c_{1}\frac{J_{4}\left(2\sqrt{z}\right)}{z^{2}}+c_{2}\frac{Y_{4}\left(2\sqrt{z}\right)}{z^{2}}+c_{3}\frac{I_{4}\left(2\sqrt{z}\right)}{z^{2}}+c_{4}\frac{K_{4}\left(2\sqrt{z}\right)}{z^{2}}\;,\\ \chi^{\prime \prime \prime }\left(z\right)=-c_{1}\frac{J_{5}\left(2\sqrt{z}\right)}{z^{5/2}}-c_{2}\frac{Y_{5}\left(2\sqrt{z}\right)}{z^{5/2}}+c_{3}\frac{I_{5}\left(2\sqrt{z}\right)}{z^{5/2}}-c_{4}\frac{K_{5}\left(2\sqrt{z}\right)}{z^{5/2}}\;, \end{array} \end{array}$$ (19)

which will be used below for application of various boundary conditions in each particular case. In the case of a full-length conical whisker, the real tip is located at *x* = 0. The Bessel functions *Y*_2_ and *K*_2_ diverge at *x* = 0 and we must put *c*_2_ = *c*_4_ = 0 (see [54] and [56]). Further, since we are interested in the fixed-base boundary conditions at *x* = *L*, *i*.*e*., *z* = *qL* ≡ *β*, we require zero deflection and zero slope at the base:

$$\begin{array}{c} \chi \left(\beta \right)=0,\;\chi^{\prime }\left(\beta \right)=0,\;c_{2}=c_{4}=0\;. \end{array}$$ (20)

These boundary conditions allow to determine the eigenfrequencies and the remaining coefficients *c*_1_, *c*_3_. Using the first derivative from Eq (19) we obtain:

$$\begin{array}{c} (\begin{array}{cc} J_{2}\left(2\sqrt{\beta }\right) & I_{2}\left(2\sqrt{\beta }\right)\\ -J_{3}\left(2\sqrt{\beta }\right) & I_{3}\left(2\sqrt{\beta }\right) \end{array})(\begin{array}{c} c_{1}\\ c_{3} \end{array})=0\;. \end{array}$$ (21)

The solution of Eq (21) exists only when the matrix determinant vanishes:

$$\begin{array}{c} J_{2}\left(2\sqrt{\beta }\right)I_{3}\left(2\sqrt{\beta }\right)+J_{3}\left(2\sqrt{\beta }\right)I_{2}\left(2\sqrt{\beta }\right)=0\;. \end{array}$$ (22)

Using the recurrence relations [55], we rewrite Eq (22) in a slightly more convenient form:

$$\begin{array}{c} J_{2}\left(2\sqrt{\beta }\right)I_{1}\left(2\sqrt{\beta }\right)-J_{1}\left(2\sqrt{\beta }\right)I_{2}\left(2\sqrt{\beta }\right)=0\;. \end{array}$$ (23)

The *j*-th root of Eq (23), *β*_*j*_, gives the eigenfrequencies of the full-length conical whisker:

$$\begin{array}{c} \omega_{j}=\frac{\beta_{j}}{2}\frac{\xi }{L}\sqrt{\frac{E}{\rho }}=\frac{\beta_{j}}{2}\frac{R}{L^{2}}\sqrt{\frac{E}{\rho }}\;, \end{array}$$ (24)

and their corresponding eigenmodes:

$$\begin{array}{c} \chi_{j}\left(z\right)=\frac{C_{j}}{z}(J_{2}\left(2\sqrt{z}\right)-\frac{J_{2}\left(2\sqrt{\beta_{j}}\right)}{I_{2}\left(2\sqrt{\beta_{j}}\right)}I_{2}\left(2\sqrt{z}\right))\;. \end{array}$$ (25)

The actual eigenmodes *X*_*j*_(*x*) (or *Z*_*j*_(*x*), see below) are then obtained from Eq (25) by replacing *χ*_*j*_(*z*) → *X*_*j*_(*x*), *z* → *qx* and *β* → *qL*. The constant *C*_*j*_ in Eq (25) is found separately for each one of the modes from the normalization condition:

$$\begin{array}{c} \int_{0}^{L}x^{2}X_{j}^{2}\left(x\right)dx=\frac{1}{q_{j}^{3}}\int_{0}^{\beta_{j}}z^{2}\chi_{j}^{2}\left(z\right)dz=1\;. \end{array}$$ (26)

The first ten dimensionless eigenfrequencies *β*_*j*_ of the freely vibrating full-length conical whisker, as given by Eq (23), are 8.719, 21.146, 38.454, 60.680, 87.834, 119.919, 156.936, 198.887, 245.771, 297.589.

#### Freely vibrating truncated conical whisker

For a freely vibrating truncated conical whisker the general solution of Eq (14) is also given by Eq (18). However, in the truncated cone case, the whisker tip is not at the origin but at *x* = *ℓ*. The whisker base is still at *x* = *L*, as before (the whisker length is then *L* − *ℓ*). We have to apply boundary conditions at both tip and base of the cone. The whisker tip is free, so the boundary conditions are vanishing bending moment and shear force at the tip *x* = *ℓ*, or, in a dimensionless form, at *z* = *qℓ* ≡ *γ*. At the base, *x* = *L* or *z* = *qL* ≡ *β*, the boundary conditions are the same as before: zero deflection and zero slope. These boundary conditions can be written as:

$$\begin{array}{ccc} \chi & = & 0,\;\frac{d\chi }{dz}=0\;\;\text{at}\;z=qL\equiv \beta \;(\text{whisker}\;\text{base})\;,\\ z^{4}\frac{d^{2}\chi }{dz^{2}} & = & 0,\;\frac{d}{dz}(z^{4}\frac{d^{2}\chi }{dz^{2}})=0\;\text{at}\;z=q\ell \equiv \gamma \;(\text{whisker}\;\text{tip})\;. \end{array}$$ (27)

Using these equations and expressions for derivatives in Eq (19), we obtain the following equation for the four coefficients *c*_1_, *c*_2_, *c*_3_, *c*_4_:

$$\begin{array}{c} (\begin{array}{cccc} J_{2}\left(2\sqrt{\beta }\right) & Y_{2}\left(2\sqrt{\beta }\right) & I_{2}\left(2\sqrt{\beta }\right) & K_{2}\left(2\sqrt{\beta }\right)\\ J_{3}\left(2\sqrt{\beta }\right) & Y_{3}\left(2\sqrt{\beta }\right) & -I_{3}\left(2\sqrt{\beta }\right) & K_{3}\left(2\sqrt{\beta }\right)\\ J_{4}\left(2\sqrt{\gamma }\right) & Y_{4}\left(2\sqrt{\gamma }\right) & I_{4}\left(2\sqrt{\gamma }\right) & K_{4}\left(2\sqrt{\gamma }\right)\\ J_{5}\left(2\sqrt{\gamma }\right) & Y_{5}\left(2\sqrt{\gamma }\right) & -I_{5}\left(2\sqrt{\gamma }\right) & K_{5}\left(2\sqrt{\gamma }\right) \end{array})(\begin{array}{c} c_{1}\\ c_{2}\\ c_{3}\\ c_{4} \end{array})=0\;. \end{array}$$ (28)

Again, using the Bessel function recurrence relations [55] we can slightly simplify the last row in the above equation:

$$\begin{array}{c} (\begin{array}{cccc} J_{2}\left(2\sqrt{\beta }\right) & Y_{2}\left(2\sqrt{\beta }\right) & I_{2}\left(2\sqrt{\beta }\right) & K_{2}\left(2\sqrt{\beta }\right)\\ J_{3}\left(2\sqrt{\beta }\right) & Y_{3}\left(2\sqrt{\beta }\right) & -I_{3}\left(2\sqrt{\beta }\right) & K_{3}\left(2\sqrt{\beta }\right)\\ J_{4}\left(2\sqrt{\gamma }\right) & Y_{4}\left(2\sqrt{\gamma }\right) & I_{4}\left(2\sqrt{\gamma }\right) & K_{4}\left(2\sqrt{\gamma }\right)\\ J_{3}\left(2\sqrt{\gamma }\right) & Y_{3}\left(2\sqrt{\gamma }\right) & I_{3}\left(2\sqrt{\gamma }\right) & -K_{3}\left(2\sqrt{\gamma }\right) \end{array})(\begin{array}{c} c_{1}\\ c_{2}\\ c_{3}\\ c_{4} \end{array})=0\;. \end{array}$$ (29)

Finally, setting the determinant of the above matrix to zero, the roots and the four coefficients *c*_1_, *c*_2_, *c*_3_, *c*_4_ can be found numerically. The corresponding eigenfrequencies of the freely vibrating truncated conical whisker are:

$$\begin{array}{c} \omega_{j}=\frac{\gamma_{j}}{2}\frac{\xi }{\ell }\sqrt{\frac{E}{\rho }}=\frac{\beta_{j}}{2}\frac{\xi }{L}\sqrt{\frac{E}{\rho }}\;. \end{array}$$ (30)

The normalization condition for the eigenmodes (see Eq (26) for comparison) is:

$$\begin{array}{c} \int_{\ell }^{L}x^{2}X_{j}^{2}\left(x\right)dx=\frac{1}{q_{j}^{3}}\int_{\gamma_{j}}^{\beta_{j}}z^{2}\chi_{j}^{2}\left(z\right)dz=1\;. \end{array}$$ (31)

The dependence of several *β*_*j*_ of the truncation length *ℓ* is shown in Fig 7b.

#### Truncated conical whisker in contact with pole

When the whisker comes in contact with pole, the eigenmodes of the free vibration described above are modified. The presence of the pole imposes additional restrictions on the vibrational motion, which can be modeled by placing an additional simple support at the point of force application, *x* = *c*, which we label by *η* = *qc* in a dimensionless form. At this simple support, the whisker is allowed to rotate while maintaining continuity of the slope and bending moment [57]. Also, its deflection from the pole must remain zero as long as the external force is applied by the pole. We write the general solution of Eq (14) separately in each one of the two regions, to the left and to the right from the pole:

$$\begin{array}{c} \begin{array}{c} \chi_{L}\left(z\right)=c_{1}\frac{J_{2}\left(2\sqrt{z}\right)}{z}+c_{2}\frac{Y_{2}\left(2\sqrt{z}\right)}{z}+c_{3}\frac{I_{2}\left(2\sqrt{z}\right)}{z}+c_{4}\frac{K_{2}\left(2\sqrt{z}\right)}{z},\;\gamma \leq z\leq \eta \\ \chi_{R}\left(z\right)=c_{5}\frac{J_{2}\left(2\sqrt{z}\right)}{z}+c_{6}\frac{Y_{2}\left(2\sqrt{z}\right)}{z}+c_{7}\frac{I_{2}\left(2\sqrt{z}\right)}{z}+c_{8}\frac{K_{2}\left(2\sqrt{z}\right)}{z},\;\eta \leq z\leq \beta \end{array} \end{array}$$ (32)

Following [53] and [57], we write the following eight boundary conditions:

- As before, at the whisker base (*x* = *L*, *β* = *qL*) both the deflection and the slope are zero:
  $$\begin{array}{c} \chi_{R}\left(\beta \right)=0\;,\;\chi_{R}^{\prime }\left(\beta \right)=0\;. \end{array}$$ (33)
- At the point of force application (*x* = *c*, *η* = *qc*), the deflection is zero, and both slope and bending moment are continuous:
  $$\begin{array}{c} \chi_{L}\left(\eta \right)=\chi_{R}\left(\eta \right)=0\;,\;\chi_{L}^{\prime }\left(\eta \right)=\chi_{R}^{\prime }\left(\eta \right)\;,\;\chi_{L}^{\prime \prime }\left(\eta \right)=\chi_{R}^{\prime \prime }\left(\eta \right)\;. \end{array}$$ (34)
- As before, at the free tip (*x* = *ℓ*, *γ* = *qℓ*), we set the bending moment and shear force to zero:
  $$\begin{array}{c} \chi_{L}^{\prime \prime }\left(\gamma \right)=0\;,\;\chi_{L}^{\prime \prime \prime }\left(\gamma \right)=0\;. \end{array}$$ (35)

The required derivatives of *χ*_*L*_(*z*) can be taken directly from Eq (19), and the derivatives of *χ*_*R*_(*z*) can then be obtained simply by the coefficient replacement *c*_1_, *c*_2_, *c*_3_, *c*_4_ → *c*_5_, *c*_6_, *c*_7_, *c*_8_.

The boundary conditions in Eqs (33)–(35) produce the following matrix equation for a truncated conical whisker touching a pole:

$$\left(\begin{array}{c} \frac{\begin{array}{cccccccc} 0 & 0 & 0 & 0 & J_{2}(2\sqrt{\beta }) & Y_{2}(2\sqrt{\beta }) & I_{2}(2\sqrt{\beta }) & K_{2}(2\sqrt{\beta })\\ 0 & 0 & 0 & 0 & -J_{3}(2\sqrt{\beta }) & -Y_{3}(2\sqrt{\beta }) & I_{3}(2\sqrt{\beta }) & -K_{3}(2\sqrt{\beta }) \end{array}}{\begin{array}{cccccccc} J_{2}(2\sqrt{\eta }) & Y_{2}(2\sqrt{\eta }) & I_{2}(2\sqrt{\eta }) & K_{2}(2\sqrt{\eta }) & 0 & 0 & 0 & 0\\ 0 & 0 & 0 & 0 & J_{2}(2\sqrt{\eta }) & Y_{2}(2\sqrt{\eta }) & I_{2}(2\sqrt{\eta }) & K_{2}(2\sqrt{\eta }) \end{array}}\\ \frac{\begin{array}{cccccccc} -J_{3}(2\sqrt{\eta }) & -Y_{3}(2\sqrt{\eta }) & I_{3}(2\sqrt{\eta }) & -K_{3}(2\sqrt{\eta }) & J_{3}(2\sqrt{\eta }) & Y_{3}(2\sqrt{\eta }) & -I_{3}(2\sqrt{\eta }) & K_{3}(2\sqrt{\eta })\\ J_{4}(2\sqrt{\eta }) & Y_{4}(2\sqrt{\eta }) & I_{4}(2\sqrt{\eta }) & K_{4}(2\sqrt{\eta }) & -J_{4}(2\sqrt{\eta }) & -Y_{4}(2\sqrt{\eta }) & -I_{4}(2\sqrt{\eta }) & -K_{4}(2\sqrt{\eta }) \end{array}}{\begin{array}{cccccccc} J_{4}(2\sqrt{\gamma }) & Y_{4}(2\sqrt{\gamma }) & I_{4}(2\sqrt{\gamma }) & K_{4}(2\sqrt{\gamma }) & 0 & 0 & 0 & 0\\ -J_{5}(2\sqrt{\gamma }) & -Y_{5}(2\sqrt{\gamma }) & I_{5}(2\sqrt{\gamma }) & -K_{5}(2\sqrt{\gamma }) & 0 & 0 & 0 & 0 \end{array}} \end{array}\right)\left(\begin{array}{c} c_{1}\\ c_{2}\\ c_{3}\\ c_{4}\\ c_{5}\\ c_{6}\\ c_{7}\\ c_{8} \end{array}\right)=0,$$ (36)

where the order of the matrix rows agrees with the order of Eqs (33)–(35). As before, setting the determinant of the matrix to zero and finding the roots provides us with the eigenfrequencies and coefficients *c*_1_, *c*_2_, …, *c*_8_ for the eigenmodes. The eigenfrequencies of a truncated conical whisker touching a pole can be written as:

$$\begin{array}{c} \omega_{j}=\frac{\gamma_{j}}{2}\frac{\xi }{\ell }\sqrt{\frac{E}{\rho }}=\frac{\beta_{j}}{2}\frac{\xi }{L}\sqrt{\frac{E}{\rho }}=\frac{\eta_{j}}{2}\frac{\xi }{c}\sqrt{\frac{E}{\rho }}\;, \end{array}$$ (37)

The normalization condition for the eigenmodes in this case becomes:

$$\begin{array}{c} \int_{\ell }^{L}x^{2}X_{j}^{2}\left(x\right)dx=\frac{1}{q_{j}^{3}}[\int_{\gamma_{j}}^{\eta_{j}}z^{2}\chi_{L}^{2}\left(z\right)dz+\int_{\eta_{j}}^{\beta_{j}}z^{2}\chi_{R}^{2}\left(z\right)dz]=1\;. \end{array}$$ (38)

#### Full-length conical whisker in contact with pole

Finally, we describe a full-length conical whisker touching a pole at *x* = *c*. The whisker tip is located at the origin, *x* = 0 (and the truncation length is zero, *ℓ* = 0). As it was shown before, this implies that the coefficients multiplying the Bessel functions *Y*_2_ and *K*_2_ in the general solution to the left from the pole must be set to zero because of the logarithmic divergence of *Y*_2_ and *K*_2_ at the origin. This leaves us with 6 independent coefficients. As before, the eigenmodes are written separately in two regions, to the left and to the right from the pole:

$$\begin{array}{c} \begin{array}{c} \chi_{L}\left(z\right)=c_{1}\frac{J_{2}\left(2\sqrt{z}\right)}{z}+c_{2}\frac{I_{2}\left(2\sqrt{z}\right)}{z},\;\;\;\;\;\;\;0\leq z\leq \eta \\ \chi_{R}\left(z\right)=c_{3}\frac{J_{2}\left(2\sqrt{z}\right)}{z}+c_{4}\frac{Y_{2}\left(2\sqrt{z}\right)}{z}+c_{5}\frac{I_{2}\left(2\sqrt{z}\right)}{z}+c_{6}\frac{K_{2}\left(2\sqrt{z}\right)}{z},\;\eta \leq z\leq \beta \end{array} \end{array}$$ (39)

Consequently, we drop the two boundary conditions at the origin (Eq (35)). These changes are equivalent to dropping the 2nd and 4th columns and the two bottom rows in the matrix in Eq (36). The set of boundary condition equations of a full-length conical whisker touching a pole can be written as:

$$\left(\frac{\begin{array}{cccccc} 0 & 0 & J_{2}(2\sqrt{\beta }) & Y_{2}(2\sqrt{\beta }) & I_{2}(2\sqrt{\beta }) & K_{2}(2\sqrt{\beta })\\ 0 & 0 & -J_{3}(2\sqrt{\beta }) & -Y_{3}(2\sqrt{\beta }) & I_{3}(2\sqrt{\beta }) & -K_{3}(2\sqrt{\beta }) \end{array}}{\begin{array}{cccccc} J_{2}(2\sqrt{\eta }) & I_{2}(2\sqrt{\eta }) & 0 & 0 & 0 & 0\\ 0 & 0 & J_{2}(2\sqrt{\eta }) & Y_{2}(2\sqrt{\eta }) & I_{2}(2\sqrt{\eta }) & K_{2}(2\sqrt{\eta })\\ -J_{3}(2\sqrt{\eta }) & I_{3}(2\sqrt{\eta }) & J_{3}(2\sqrt{\eta }) & Y_{3}(2\sqrt{\eta }) & -I_{3}(2\sqrt{\eta }) & K_{3}(2\sqrt{\eta })\\ J_{4}(2\sqrt{\eta }) & I_{4}(2\sqrt{\eta }) & -J_{4}(2\sqrt{\eta }) & -Y_{4}(2\sqrt{\eta }) & -I_{4}(2\sqrt{\eta }) & -K_{4}(2\sqrt{\eta }) \end{array}}\right)\left(\begin{array}{c} c_{1}\\ c_{2}\\ c_{3}\\ c_{4}\\ c_{5}\\ c_{6} \end{array}\right)=0.$$ (40)

The eigenmodes and eigenfrequencies can be found numerically by the procedure described above. The normalization condition for the eigenmodes is given in Eq (38). The dependence of several conical whisker eigenfrequencies on the pole position *c* is shown in Fig 7a. One can see that the pole position strongly affects the frequencies.

#### Orthogonality of conical eigenmodes of a free whisker

First, we show the orthogonality of different eigenmodes of a freely vibrating conical whisker. Let us write Eq (11) for two conical eigenmodes *X*_*i*_(*x*) and *X*_*j*_(*x*) having angular frequencies *ω*_*i*_ and *ω*_*j*_:

$$\begin{array}{ccc} \frac{\partial^{2}}{\partial x^{2}}(x^{4}\frac{\partial^{2}X_{i}}{\partial x^{2}}) & = & \frac{4\rho \omega_{i}^{2}}{E\xi^{2}}x^{2}X_{i}\left(x\right)\;,\\ \frac{\partial^{2}}{\partial x^{2}}(x^{4}\frac{\partial^{2}X_{j}}{\partial x^{2}}) & = & \frac{4\rho \omega_{j}^{2}}{E\xi^{2}}x^{2}X_{j}\left(x\right)\;. \end{array}$$ (41)

Multiplying each of the equations by the other eigenmode and integrating we obtain:

$$\begin{array}{ccc} \int_{\ell }^{L}X_{j}\frac{\partial^{2}}{\partial x^{2}}(x^{4}\frac{\partial^{2}X_{i}}{\partial x^{2}})dx & = & \frac{4\rho \omega_{i}^{2}}{E\xi^{2}}\int_{\ell }^{L}X_{j}\left(x\right)x^{2}X_{i}\left(x\right)dx\;,\\ \int_{\ell }^{L}X_{i}\frac{\partial^{2}}{\partial x^{2}}(x^{4}\frac{\partial^{2}X_{j}}{\partial x^{2}})dx & = & \frac{4\rho \omega_{j}^{2}}{E\xi^{2}}\int_{\ell }^{L}X_{i}\left(x\right)x^{2}X_{j}\left(x\right)dx\;. \end{array}$$ (42)

By subtracting the first equation from the second we get:

$$\begin{array}{c} \begin{array}{cc} \int_{\ell }^{L}X_{j}\frac{\partial^{2}}{\partial x^{2}} & (x^{4}\frac{\partial^{2}X_{i}}{\partial x^{2}})dx-\int_{\ell }^{L}X_{i}\frac{\partial^{2}}{\partial x^{2}}(x^{4}\frac{\partial^{2}X_{j}}{\partial x^{2}})dx=\\ & =\frac{4\rho }{E\xi^{2}}\left(\omega_{i}^{2}-\omega_{j}^{2}\right)\int_{\ell }^{L}X_{i}\left(x\right)x^{2}X_{j}\left(x\right)dx\;. \end{array} \end{array}$$ (43)

Integrating by parts twice the first term on the left-hand side of Eq (43) we obtain:

$$\begin{array}{c} \begin{array}{cc} \int_{\ell }^{L} & X_{j}\frac{\partial^{2}}{\partial x^{2}}(x^{4}\frac{\partial^{2}X_{i}}{\partial x^{2}})dx=X_{j}\frac{\partial }{\partial x}(x^{4}\frac{\partial^{2}X_{i}}{\partial x^{2}}){|}_{\ell }^{L}-\int_{\ell }^{L}\frac{\partial X_{j}}{\partial x}\frac{\partial }{\partial x}(x^{4}\frac{\partial^{2}X_{i}}{\partial x^{2}})dx\\ & =X_{j}\frac{\partial }{\partial x}(x^{4}\frac{\partial^{2}X_{i}}{\partial x^{2}}){|}_{\ell }^{L}-\frac{\partial X_{j}}{\partial x}(x^{4}\frac{\partial^{2}X_{i}}{\partial x^{2}}){|}_{\ell }^{L}+\int_{\ell }^{L}\frac{\partial^{2}X_{j}}{\partial x^{2}}(x^{4}\frac{\partial^{2}X_{i}}{\partial x^{2}})dx\;. \end{array} \end{array}$$ (44)

For boundary conditions considered in this paper, that is fixed-base at *x* = *L* and free-tip at *x* = *ℓ* (Eqs (20) or (27)), the first two terms vanish, resulting in:

$$\begin{array}{c} \int_{\ell }^{L}X_{j}\frac{\partial^{2}}{\partial x^{2}}(x^{4}\frac{\partial^{2}X_{i}}{\partial x^{2}})dx=\int_{\ell }^{L}\frac{\partial^{2}X_{j}}{\partial x^{2}}(x^{4}\frac{\partial^{2}X_{i}}{\partial x^{2}})dx\;. \end{array}$$ (45)

The same result holds for the second term on the left-hand side of Eq (43), which can be obtained by merely interchanging the indices *i* and *j* in Eq (45). This means that the left-hand side of Eq (43) vanishes and we arrive at:

$$\begin{array}{c} \frac{4\rho }{E\xi^{2}}\left(\omega_{i}^{2}-\omega_{j}^{2}\right)\int_{\ell }^{L}X_{i}\left(x\right)x^{2}X_{j}\left(x\right)dx=0\;. \end{array}$$ (46)

The eigenfrequencies of two different eigenmodes, *i* ≠ *j*, are not equal, *ω*_*i*_ ≠ *ω*_*j*_, and we get the first orthogonality condition:

$$\begin{array}{c} \int_{\ell }^{L}X_{i}\left(x\right)x^{2}X_{j}\left(x\right)dx=0,\;i\neq j\;. \end{array}$$ (47)

Further, any of Eq (42) gives the second orthogonality condition:

$$\begin{array}{c} \int_{\ell }^{L}X_{j}\frac{\partial^{2}}{\partial x^{2}}(x^{4}\frac{\partial^{2}X_{i}}{\partial x^{2}})dx=0,\;i\neq j\;. \end{array}$$ (48)

Using the normalization condition $\int_{\ell }^{L}x^{2}X_{i}^{2}\left(x\right)dx=1$, and Eq (42) with *i* = *j*, we obtain:

$$\begin{array}{c} \int_{\ell }^{L}X_{i}\frac{\partial^{2}}{\partial x^{2}}(x^{4}\frac{\partial^{2}X_{i}}{\partial x^{2}})dx=\frac{4\rho \omega_{i}^{2}}{E\xi^{2}}\;. \end{array}$$ (49)

To summarize, we can write the two useful orthogonality properties of the vibrational eigenmodes as:

$$\begin{array}{ccc} \int_{\ell }^{L}X_{i}\left(x\right)x^{2}X_{j}\left(x\right)dx & = & \delta_{ij}\;,\\ \int_{\ell }^{L}X_{i}\frac{\partial^{2}}{\partial x^{2}}(x^{4}\frac{\partial^{2}X_{j}}{\partial x^{2}})dx & = & \frac{4\rho \omega_{i}^{2}}{E\xi^{2}}\delta_{ij}\;, \end{array}$$ (50)

where *δ*_*ij*_ is the Kronecker delta.

#### Orthogonality of conical eigenmodes of a whisker in contact with pole

Now we show that different eigenmodes of a conical whisker touching a pole are orthogonal as well. As in the free whisker case, we write Eq (11) for two different conical eigenmodes *X*_*i*_(*x*) and *X*_*j*_(*x*), multiply each of the equations by the other eigenmode, integrate, and subtract one equation from another (analogously to Eqs (41)–(43)):

$$\begin{array}{c} \begin{array}{cc} \int_{\ell }^{L}X_{j}\frac{\partial^{2}}{\partial x^{2}} & (x^{4}\frac{\partial^{2}X_{i}}{\partial x^{2}})dx-\int_{\ell }^{L}X_{i}\frac{\partial^{2}}{\partial x^{2}}(x^{4}\frac{\partial^{2}X_{j}}{\partial x^{2}})dx=\\ & =\frac{4\rho }{E\xi^{2}}\left(\omega_{i}^{2}-\omega_{j}^{2}\right)\int_{\ell }^{L}X_{i}\left(x\right)x^{2}X_{j}\left(x\right)dx\;. \end{array} \end{array}$$ (51)

As the whisker is now piecewise described by two functions *X*_*L*,*i*_(*x*) and *X*_*R*,*i*_(*x*) (to the left and to the right from the pole at *x* = *c*), the integration should be split into two corresponding intervals, *ℓ* < *x* < *c* and *c* < *x* < *L*, for example:

$$\begin{array}{c} \int_{\ell }^{L}X_{j}\frac{\partial^{2}}{\partial x^{2}}(x^{4}\frac{\partial^{2}X_{i}}{\partial x^{2}})dx=\int_{\ell }^{c}X_{j}\frac{\partial^{2}}{\partial x^{2}}(x^{4}\frac{\partial^{2}X_{i}}{\partial x^{2}})dx+\int_{c}^{L}X_{j}\frac{\partial^{2}}{\partial x^{2}}(x^{4}\frac{\partial^{2}X_{i}}{\partial x^{2}})dx\;. \end{array}$$ (52)

Integrating by parts twice the first term in Eq (51) and noting that the terms at *x* = *ℓ* and *x* = *L* vanish (as in Eq (44)), we get:

$$\begin{array}{c} \begin{array}{cc} \int_{\ell }^{L} & X_{j}\frac{\partial^{2}}{\partial x^{2}}(x^{4}\frac{\partial^{2}X_{i}}{\partial x^{2}})dx=X_{L,j}\frac{\partial }{\partial x}(x^{4}\frac{\partial^{2}X_{L,i}}{\partial x^{2}}){|}_{x=c}-\frac{\partial X_{L,j}}{\partial x}(x^{4}\frac{\partial^{2}X_{L,i}}{\partial x^{2}}){|}_{x=c}\\ & -X_{R,j}\frac{\partial }{\partial x}(x^{4}\frac{\partial^{2}X_{R,i}}{\partial x^{2}}){|}_{x=c}+\frac{\partial X_{R,j}}{\partial x}(x^{4}\frac{\partial^{2}X_{R,i}}{\partial x^{2}}){|}_{x=c}+\int_{\ell }^{L}\frac{\partial^{2}X_{j}}{\partial x^{2}}(x^{4}\frac{\partial^{2}X_{i}}{\partial x^{2}})dx\;. \end{array} \end{array}$$ (53)

The boundary conditions at the pole, Eq (34), require zero dispacement and continuity of the first and second derivatives at *x* = *c*. Therefore, the four terms evaluated at *x* = *c* vanish, resulting in:

$$\begin{array}{c} \int_{\ell }^{L}X_{j}\frac{\partial^{2}}{\partial x^{2}}(x^{4}\frac{\partial^{2}X_{i}}{\partial x^{2}})dx=\int_{\ell }^{L}\frac{\partial^{2}X_{j}}{\partial x^{2}}(x^{4}\frac{\partial^{2}X_{i}}{\partial x^{2}})dx\;. \end{array}$$ (54)

This equation is identical to Eq (45) we obtained above for a freely vibrating whisker. Following the same reasoning as in transition from Eqs (45) to (50), we conclude that the orthogonality properties of the vibrational eigenmodes of a whisker in contact with pole are identical to those of a freely vibrating whisker as given in Eq (50).

### Time dependence of the whisker motion

#### Whisker motion in contact with pole

The time dependent shape of the whisker subject to external forces and damping is described by the following inhomogeneous differential equation in the small angle approximation [5, 53]:

$$\begin{array}{c} \frac{\partial^{2}}{\partial x^{2}}(EI\left(x\right)\frac{\partial^{2}y}{\partial x^{2}})+\mu \left(x\right)\frac{\partial^{2}y}{\partial t^{2}}+\mu \left(x\right)\alpha \frac{\partial y}{\partial t}=f\left(x,t\right)\;, \end{array}$$ (55)

where *y* = *y*(*x*, *t*) is the total displacement of the whisker from *x*-axis, *E* is the Young’s modulus, *α* is the frequency-independent viscous damping coefficient, *I*(*x*) and *μ*(*x*) are given in Eq (9), and *f*(*x*, *t*) is the linear density of applied force. In comparison to the corresponding homogeneous eigenmode Eq (8), the generalized Eq (55) contains a linear damping term and an inhomogeneous term describing the external force. For a point force applied by pole at *x* = *c*, we can write *f*(*x*, *t*) = *F*(*t*)*δ*(*x* − *c*), where *δ*(*x*) is the Dirac delta function. To find the vibration of the whisker induced by its interactions with an object, such as the poles used in behavioral experiments [58], we decompose the solution, similar to [24], into a steady state displacement, *y*_*s*_(*x*, *t*), and a vibrational contribution, *y*_*e*_(*x*, *t*), as *y*(*x*, *t*) = *y*_*s*_(*x*, *t*) + *y*_*e*_(*x*, *t*). No approximation is made in decomposing the solution in this way.

First, we need to obtain the steady state term, *y*_*s*_(*x*, *t*), in response to the time varying force *F*(*t*). This term satisfies the following steady state equation:

$$\begin{array}{c} \frac{\partial^{2}}{\partial x^{2}}(EI\left(x\right)\frac{\partial^{2}y_{s}}{\partial x^{2}})=F\left(t\right)\delta \left(x-c\right)\;. \end{array}$$ (56)

This equation is equivalent to Eq (1), which describes steady state bending under a point force *F*. Eq (56) is obtained by slightly rearranging Eq (1), substituting *M*(*x*) from Eq (2), and differentiating twice. The solution of both Eqs (1) and (56) is described in Eqs (3)–(7). One can see that in the small angle approximation the steady state deflection of the whisker is directly proportional to the applied force *F*(*t*), so we can write *y*_*s*_ in a factorized form:

$$\begin{array}{c} y_{s}\left(x,t\right)=F\left(t\right){\tilde{y}}_{s}\left(x\right)\;. \end{array}$$ (57)

Substituting the general form of the solution *y*(*x*, *t*) = *y*_*s*_(*x*, *t*) + *y*_*e*_(*x*, *t*), with $y_{s}\left(x,t\right)=F\left(t\right){\tilde{y}}_{s}\left(x\right)$, into Eq (55), we arrive at the following equation for the vibrational term *y*_*e*_(*x*, *t*):

$$\begin{array}{c} \frac{\partial^{2}}{\partial x^{2}}(EI\left(x\right)\frac{\partial^{2}y_{e}}{\partial x^{2}})+\mu \left(x\right)\frac{\partial^{2}y_{e}}{\partial t^{2}}+\alpha \mu \left(x\right)\frac{\partial y_{e}}{\partial t}=-\mu \left(x\right)[\ddot{F}\left(t\right)+\alpha \dot{F}\left(t\right)]{\tilde{y}}_{s}\left(x\right)\;, \end{array}$$ (58)

where the over-dots indicate time derivatives. The standard technique [59] is used to solve this inhomogeneous equation for *y*_*e*_(*x*, *t*). We use the vibrational eigenmodes, which were found analytically above by solving the corresponding homogeneous equation, as a basis to find the time-dependent solutions of the inhomogeneous Eq (58). Following [59], we expand the solution of Eq (58) as:

$$\begin{array}{c} y_{e}\left(x,t\right)=\sum_{j}\phi_{j}\left(t\right)X_{j}\left(x\right)\;, \end{array}$$ (59)

where *ϕ*_*j*_(*t*) are time-dependent functions to be found. Substituting this expansion into Eq (58), multiplying by one of the spatial eigenmodes, integrating over *x*, and using the orthogonality of the vibrational eigenmodes in Eq (50), we obtain the following equation for the time-dependent expansion coefficients *ϕ*_*j*_(*t*):

$$\begin{array}{c} {\ddot{\phi }}_{j}\left(t\right)+\alpha {\dot{\phi }}_{j}\left(t\right)+\omega_{j}^{2}\phi_{j}\left(t\right)=-[\ddot{F}\left(t\right)+\alpha \dot{F}\left(t\right)]P_{j}\;, \end{array}$$ (60)

where *ω*_*j*_ is the angular frequency of the *j*-th eigenmode and $P_{j}=\int_{\ell }^{L}{\tilde{y}}_{s}\left(x\right)x^{2}X_{j}\left(x\right)dx$. In the under-damped case, *ω*_*j*_ > *α*/2, the solution of the inhomogeneous Eq (60) can be found using standard techniques [60]:

$$\begin{array}{c} \phi_{j}\left(t\right)=-\frac{P_{j}}{{\tilde{\omega }}_{j}}\int_{0}^{t}[\ddot{F}\left(t^{\prime }\right)+\alpha \dot{F}\left(t^{\prime }\right)]e^{-\frac{\alpha }{2}\left(t-t^{\prime }\right)}\;\text{sin}\;({\tilde{\omega }}_{j}\left(t-t^{\prime }\right))dt^{\prime }\;, \end{array}$$ (61)

where ${\tilde{\omega }}_{j}=\sqrt{\omega_{j}^{2}-{\left(\alpha /2\right)}^{2}}$. The integrals in Eq (61) can be easily evaluated numerically for any force profile *F*(*t*) in question. The full solution describing the whisker motion under applied force *F*(*t*) can then be written as:

$$\begin{array}{c} y\left(x,t\right)=F\left(t\right){\tilde{y}}_{s}\left(x\right)+\sum_{j}\phi_{j}\left(t\right)X_{j}\left(x\right)\;. \end{array}$$ (62)

#### Whisker motion after detachment from pole

We also need to describe the whisker motion after its detachment from the pole. Obviously, after whisker detachment the external force vanishes and the whisker behavior is determined by Eq (55) with *f*(*x*, *t*) ≡ 0. In the case of such a free motion, instead of decomposing the solution into steady state and vibrational parts, we seek the full solution of Eq (55), which we label by *u*(*x*, *t*), as an expansion in the free-motion spatial eigenmodes *Z*_*j*_(*x*) of the fixed-base free-tip conical whisker:

$$\begin{array}{c} u\left(x,t\right)=\sum_{j}\varphi_{j}\left(t\right)Z_{j}\left(x\right)\;. \end{array}$$ (63)

As before, the eigenmodes *Z*_*j*_(*x*) of the homogeneous version of Eq (55) with zero damping are found analytically. Substituting this expansion into the equation of motion, multiplying by one of the modes, integrating, and using orthogonality relations in Eq (50) we obtain (see Eq (60) for comparison):

$$\begin{array}{c} {\ddot{\varphi }}_{j}\left(t\right)+\alpha {\dot{\varphi }}_{j}\left(t\right)+\varpi_{j}^{2}\varphi_{j}\left(t\right)=0\;, \end{array}$$ (64)

where $\varpi_{j}$ is the angular frequency of the eigenmode *Z*_*j*_(*x*). It is important to note that the free motion vibrational eigenmodes *Z*_*j*_(*x*) and their corresponding frequencies $\varpi_{j}$ are different from the eigenmodes *X*_*j*_(*x*) and frequencies *ω*_*j*_ that were found for a whisker touching the pole.

In our under-damped case, $\varpi_{j}>\alpha /2$, the solution of Eq (64) can be written as:

$$\begin{array}{c} \varphi_{j}\left(t\right)=A_{j}e^{k_{j}^{+}\left(t-t_{f}\right)}+B_{j}e^{k_{j}^{-}\left(t-t_{f}\right)}, \end{array}$$ (65)

where $k_{j}^{\pm }=-\alpha /2\pm i{\tilde{\varpi }}_{j}$, and where ${\tilde{\varpi }}_{j}=\sqrt{\varpi_{j}^{2}-{\left(\alpha /2\right)}^{2}}$. The coefficients *A*_*j*_ and *B*_*j*_ for each one of the eigenmodes are determined by the initial conditions of continuity of the whisker deflection and velocity at the instant of detachment, *t* = *t*_*f*_, at each point of the whisker, *ℓ* ≤ *x* ≤ *L*. These continuity conditions can be written as:

$$\begin{array}{c} \begin{array}{c} y(x,t_{f})=u\left(x,t_{f}\right)=\sum_{j}\varphi_{j}\left(t_{f}\right)Z_{j}\left(x\right)\;,\\ \dot{y}\left(x,t_{f}\right)=\dot{u}\left(x,t_{f}\right)=\sum_{j}{\dot{\varphi }}_{j}\left(t_{f}\right)Z_{j}\left(x\right)\;, \end{array} \end{array}$$ (66)

where *y*(*x*, *t*) describes the whisker motion at times *t* ≤ *t*_*f*_ (see Eq (62)), and *u*(*x*, *t*) describes the motion at *t* ≥ *t*_*f*_ (see Eq (63)). Projecting each one of Eq (66) on one of the eigenmodes, and using their orthogonality, we can write:

$$\begin{array}{c} \begin{array}{c} \varphi_{j}\left(t_{f}\right)=\int_{\ell }^{L}Z_{j}\left(x\right)x^{2}y\left(x,t_{f}\right)dx\;,\\ {\dot{\varphi }}_{j}\left(t_{f}\right)=\int_{\ell }^{L}Z_{j}\left(x\right)x^{2}\dot{y}\left(x,t_{f}\right)dx\;. \end{array} \end{array}$$ (67)

The time derivative of *y*(*x*, *t*) is given by:

$$\begin{array}{c} \dot{y}\left(x,t\right)=\dot{F}\left(t\right){\tilde{y}}_{s}\left(x\right)+\sum_{j}{\dot{\phi }}_{j}\left(t\right)X_{j}\left(x\right)\;, \end{array}$$ (68)

and that of *ϕ*_*j*_(*t*):

$$\begin{array}{c} {\dot{\phi }}_{j}\left(t\right)=-P_{j}\int_{0}^{t}(\ddot{F}\left(t^{\prime }\right)+\alpha \dot{F}\left(t^{\prime }\right))e^{-\frac{\alpha }{2}\left(t-t^{\prime }\right)}[\text{cos}({\tilde{\omega }}_{j}\left(t-t^{\prime }\right))-\frac{\alpha }{2{\tilde{\omega }}_{j}}\;\text{sin}\;({\tilde{\omega }}_{j}\left(t-t^{\prime }\right))]dt^{\prime }\;. \end{array}$$ (69)

Finally, combining Eqs (67) with (65) at *t* = *t*_*f*_, we find:

$$\begin{array}{c} A_{j}+B_{j}=\varphi_{j}\left(t_{f}\right)\;,\;\;k_{j}^{+}A_{j}+k_{j}^{-}B_{j}={\dot{\varphi }}_{j}\left(t_{f}\right)\;, \end{array}$$ (70)

resulting in:

$$\begin{array}{c} A_{j}=\frac{k_{j}^{-}\varphi_{j}\left(t_{f}\right)-{\dot{\varphi }}_{j}\left(t_{f}\right)}{k_{j}^{-}-k_{j}^{+}}\;,\;\;B_{j}=\frac{{\dot{\varphi }}_{j}\left(t_{f}\right)-k_{j}^{+}\varphi_{j}\left(t_{f}\right)}{k_{j}^{-}-k_{j}^{+}}\;. \end{array}$$ (71)

Substituting *A*_*j*_ and *B*_*j*_ into Eq (65), we obtain expression for the time-dependent coefficients describing the whisker dynamics after its detachment from the pole at *t* ≥ *t*_*f*_:

$$\begin{array}{c} \begin{array}{cc} \varphi_{j}\left(t\right)=e^{-\frac{\alpha }{2}\left(t-t_{f}\right)}[\varphi_{j}\left(t_{f}\right) & \left(\frac{\alpha }{2{\tilde{\varpi }}_{j}}\;\text{sin}\;({\tilde{\varpi }}_{j}\left(t-t_{f}\right))+\text{cos}({\tilde{\varpi }}_{j}\left(t-t_{f}\right))\right)\\ & +\frac{{\dot{\varphi }}_{j}\left(t_{f}\right)}{{\tilde{\varpi }}_{j}}\;\text{sin}\;({\tilde{\varpi }}_{j}\left(t-t_{f}\right))]\;, \end{array} \end{array}$$ (72)

with *φ*_*j*_(*t*_*f*_) and ${\dot{\varphi }}_{j}\left(t_{f}\right)$ given in Eq (67).

#### Time-dependent forces at the whisker base

Rodents extract the structure of the tactile world through forces applied to mechanosensors embedded in the follicle. Although the precise mechanism by which these forces excite primary sensory neurons has yet to be fully elucidated, we investigate two classes of vibrational cues rodents may use. These are the bending moment, *M*(*x* = *L*; *t*) = *EI*_0_ *y*″(*L*; *t*), and the shear force, *V*(*x* = *L*; *t*) = *M*′(*L*; *t*), at the whisker base. Here, *I*_0_ = *I*(*L*) = (*π*/4)*R*^4^, where *R* is the whisker base radius. During the force application, 0 ≤ *t* ≤ *t*_*f*_, we can find the bending moment and shear force at the whisker follicle using the expression for *y*(*t*, *x*) in Eq (62). For the bending moment we get:

$$\begin{array}{c} M\left(L;t\right)=F\left(t\right)\left(L-c\right)+EI_{0}\sum_{j}\phi_{j}\left(t\right)X_{j}^{\prime \prime }\left(L\right)\;, \end{array}$$ (73)

where the first and second terms are contributions of the static and vibrational components to the total bending moment, respectively. Analogously, for the shear force at the follicle we obtain:

$$\begin{array}{c} V\left(L;t\right)=F\left(t\right)+EI_{0}\sum_{j}\phi_{j}\left(t\right)(\frac{4}{L}X_{j}^{\prime \prime }\left(L\right)+X_{j}^{\prime \prime \prime }\left(L\right))\;, \end{array}$$ (74)

where, again, the two terms are contributions of the steady state and vibrational components.

After the detachment from pole, *t* > *t*_*f*_, the time dependence of the bending moment and the shear force can be written by using the function *u*(*x*, *t*) of the free vibrational motion in Eq (63):

$$\begin{array}{c} \begin{array}{c} M(L;t)=EI_{0}\sum_{j}\varphi_{j}\left(t\right)Z_{j}^{\prime \prime }\left(L\right)\;,\\ V(L;t)=EI_{0}\sum_{j}\varphi_{j}\left(t\right)(\frac{4}{L}Z_{j}^{\prime \prime }\left(L\right)+Z_{j}^{\prime \prime \prime }\left(L\right))\;. \end{array} \end{array}$$ (75)

The derivatives of the spatial eigenmodes in Eqs (73)–(75) can be obtained from the analytical expressions of *X*_*j*_(*x*) and *Z*_*j*_(*x*) using Bessel function recurrence relations, as in Eq (19), and using Eqs (12) and (13).

#### Gaussian force with smooth onset

Here we model the time-dependent profile of the force, *F*(*t*), applied to the whisker by pole at the point *x* = *c*. The force is acting during the time interval 0 ≤ *t* ≤ *t*_*f*_, and we assume that its time-dependence is described by the upper part of a Gaussian. At the instant of contact *t* = 0, to avoid discontinuity and/or singularity in $\dot{F}\left(t\right)$ and $\ddot{F}\left(t\right)$ entering Eq (61), we insert a smooth connecting function *F*_*s*_(*t*) into the onset of the Gaussian curve *F*_*G*_(*t*) at short times 0 ≤ *t* ≤ *τ*, where *τ* ≪ *t*_*f*_:

$$\begin{array}{c} F\left(t\right)=\{\begin{array}{cc} F_{s}\left(t\right) & 0\leq t\leq \tau \\ F_{G}\left(t\right) & \tau \leq t\leq t_{f} \end{array}\;. \end{array}$$ (76)

At the end of the force application, *t* = *t*_*f*_, the pole detaches from the whisker with finite velocity, proportional to $\dot{F}\left(t_{f}\right)$, without decelerating the whisker.

We write the truncated Gaussian profile of the force in the time interval *τ* ≤ *t* ≤ *t*_*f*_ as:

$$\begin{array}{c} F_{G}\left(t\right)=\frac{F_{\text{max}}}{1-C}\left\{\text{exp}\left[-{\left(\frac{t-a}{b}\right)}^{2}\right]-C\right\}, \end{array}$$ (77)

where *F*_max_ is the force maximum and 0 < *C* < 1 is the Gaussian cutoff. For the uncut Gaussian shape, *C* → 0. The parameters *a* and *b* are found as follows. First, from the condition of vanishing of *F*_*G*_(*t*) at *t* = *t*_*f*_ we get the relation between *a* and *b*:

$$\begin{array}{c} F_{G}\left(t_{f}\right)=0\;\Rightarrow \;b=\frac{t_{f}-a}{\sqrt{\text{ln}(1/C)}}\;. \end{array}$$ (78)

Then, the implicit equation *F*_*s*_(*τ*) = *F*_*G*_(*τ*), which ensures continuity of the force at *t* = *τ*, is solved for the remaining parameter *a*.

The smooth connecting function *F*_*s*_(*t*) describing the onset of the force at short times 0 ≤ *t* ≤ *τ*, is constructed to satisfy the following conditions: (i) $F_{s}\left(0\right)={\dot{F}}_{s}\left(0\right)={\ddot{F}}_{s}\left(0\right)=0$, (ii) *F*_*s*_(*τ*) = *F*_*G*_(*τ*), (iii) ${\dot{F}}_{s}\left(\tau \right)={\dot{F}}_{G}\left(\tau \right)$, (iv) ${\ddot{F}}_{s}\left(\tau \right)={\ddot{F}}_{G}\left(\tau \right)$, and (v) *F*_*s*_(*t*) is continuous and at least twice differentiable in the interval 0 ≤ *t* ≤ *τ*. The function that satisfies these conditions can be taken as:

$$F_{s}\left(t\right)=\frac{\tau }{3}\left[3{\dot{F}}_{G}\left(\tau \right)-\tau {\ddot{F}}_{G}\left(\tau \right)\right]{\left(\frac{t}{\tau }\right)}^{3}+\frac{\tau }{4}\left[\tau {\ddot{F}}_{G}\left(\tau \right)-2{\dot{F}}_{G}\left(\tau \right)\right]{\left(\frac{t}{\tau }\right)}^{4}.$$ (79)

Specifically, at *t* = *τ* we have:

$$\begin{array}{c} F_{s}\left(\tau \right)=\frac{\tau }{2}{\dot{F}}_{G}\left(\tau \right)-\frac{\tau^{2}}{12}{\ddot{F}}_{G}\left(\tau \right)\;. \end{array}$$ (80)

In Eqs (61), (69) and (79) we also use the first two derivatives of *F*_*G*_(*t*) and *F*_*s*_(*t*), which are:

$$\begin{array}{l} {\dot{F}}_{G}\left(t\right)=-\frac{2F_{max}}{b\left(1-C\right)}\left(\frac{t-a}{b}\right)\;\text{exp}\left[-{\left(\frac{t-a}{b}\right)}^{2}\right],\\ {\ddot{F}}_{G}\left(t\right)=\frac{2F_{max}}{b^{2}\left(1-C\right)}\left[2{\left(\frac{t-a}{b}\right)}^{2}-1]\;\text{exp}[-{\left(\frac{t-a}{b}\right)}^{2}\right], \end{array}$$ (81)

and:

$$\begin{array}{l} {\dot{F}}_{s}\left(t\right)=\left[3{\dot{F}}_{G}\left(\tau \right)-\tau {\ddot{F}}_{G}\left(\tau \right)\right]{\left(\frac{t}{\tau }\right)}^{2}+\left[\tau {\ddot{F}}_{G}\left(\tau \right)-2{\dot{F}}_{G}\left(\tau \right)\right]{\left(\frac{t}{\tau }\right)}^{3},\\ {\ddot{F}}_{s}\left(t\right)=\frac{2}{\tau }\left[3{\dot{F}}_{G}\left(\tau \right)-\tau {\ddot{F}}_{G}\left(\tau \right)]\left(\frac{t}{\tau }\right)+\frac{3}{\tau }[\tau {\ddot{F}}_{G}\left(\tau \right)-2{\dot{F}}_{G}\left(\tau \right)\right]{\left(\frac{t}{\tau }\right)}^{2}. \end{array}$$ (82)

#### Wave propagation upon impact with pole

When the whisker rapidly hits the pole, waves propagating along the whisker are created. This phenomenon was described by [24], where the following form of the force was assumed:

$$\begin{array}{c} F\left(t\right)=\{\begin{array}{cc} 0 & t<0\\ st & t\geq 0\;. \end{array} \end{array}$$ (83)

This corresponds to the whisker being stationary before the application of the force. Once the force is applied, following an instantaneous infinite acceleration at *t* = 0, the whisker starts bending at a constant rate proportional to *s*. In this case the derivatives are:

$$\begin{array}{c} \dot{F}\left(t\right)=s\Theta \left(t\right)\;,\;\ddot{F}\left(t\right)=s\delta \left(t\right)\;, \end{array}$$ (84)

where Θ(*t*) is the unit step function. Substitution of $\dot{F}\left(t\right)$ and $\ddot{F}\left(t\right)$ into Eq (61) gives:

$$\begin{array}{c} \phi_{j}\left(t\right)=-\frac{sP_{j}}{{\tilde{\omega }}_{j}}[e^{-\frac{\alpha }{2}t}\;\text{sin}\;{\tilde{\omega }}_{j}t+\alpha \int_{0}^{t}e^{-\frac{\alpha }{2}\left(t-t^{\prime }\right)}\;\text{sin}\;{\tilde{\omega }}_{j}\left(t-t^{\prime }\right)dt^{\prime }]\;. \end{array}$$ (85)

One can see from Eq (85) that a rapid application of force to the whisker results in excitation of multiple eigenmodes, and the amplitude of their excitation decreases slowly, as $1/{\tilde{\omega }}_{j}$, with the mode number, *j*. Simultaneous excitation of multiple eigenmodes results in propagating waves, as was shown by [24].

In our model, the rapidity of force application is controlled at short times by the smooth connecting function *F*_*s*_(*t*) defined in Eq (79). This function helps avoid singularities as it ensures that the applied force and its first two derivatives are continuous. The duration *τ* of this connecting function is a critical parameter characterizing the rapidity of force application. In the limit *τ* → 0, the force profile *F*(*t*) reduces to Eq (83), at least for the part of the Gaussian, *F*_*G*_(*t*), that is approximately linear at early times. The calculations show that eigenmodes with period 2*π*/*ω*_*j*_ longer than *τ* are effectively excited upon hitting the pole, while the excitation of higher eigenmodes becomes increasingly less efficient. The results of these calculations are shown in Fig 5.

### Measurement of whisker geometry and dynamics

Details of the object localization task, behavioral apparatus, high-speed videography, whisker tracking and force calculation have been described elsewhere [18, 58, 61, 62]. Head-fixed mice were trimmed to a single C2 whisker for behavioral experiments. Using this whisker they localized a steel pole (class ZZ gage pin, Vermont Gage, diameter 0.5 mm) placed in one of five positions on the anterior-posterior axis, licking for a water reward if the pole was in one of the four anterior positions. Radial distance of the pole ranged from 6.7–12.9mm from the follicle.

Backlit (940nm IR LED) whiskers were imaged from above at 1000 or 4000 fps, (90–150 *μs* exposure) using a Basler 504k or Basler Ace acA2000-340km camera, digitized and tracked using the Janelia Whisker Tracker [62]. Dual-perspective imaging was performed by projecting two orthogonal views onto a single Basler camera via mirrors. Curvature measurements at 5–6mm from the follicle base were used to calculate the steady state bending moment assuming an Euler-Bernoulli quasi-static approximation for whisker bending in a single plane, see Eq (3) [16]. Contact detection was performed automatically via custom MATLAB scripts using a threshold based on whisker curvature and distance from whisker to pole, followed by manual correction. In some experiments we tracked a piece of dust or another imperfection on the whisker (Fig 1a, arrow). This allowed us to estimate movement of the whisker into the face in response to applied axial force.

To measure whisker vibration during 4000fps imaging, we fit traced whisker coordinates from the Janelia Whisker Tracker with a fifth order polynomial. To prevent distortions by mistracking near fur or pole shadow, we excluded the 2 percent of points closest to follicle and pole from the fit. We then computed the displacement along the y-axis at 8 evenly spaced points along the fitted curve for twenty timepoints (0.25ms step size). The time of the first frame with contact was defined as 0.125ms from touch start. We extracted vibrational displacement from quasi steady-state displacement by subtracting a 3rd order polynomial fit of displacement along the anteroposterior axis over time, defining vibration to be the residual.

Following behavioral sessions, the C2 whiskers were plucked and photographed at 6.3x magnification under a light macroscope. Whisker length and width was determined using ImageJ and the NeuronJ plugin. Total length includes the portion of the whisker embedded in the follicle. Whiskers were then weighed on a microgram balance.

### Statistics and fitting

Time-dependent forces applied by the pole during active whisking were modeled by the top half of a gaussian distribution *F*(*t*) = *F*_max_{exp[−(*t* − 2*t*_*d*_)^2^/(1.2011*t*_*d*_)^2^] − 0.5} for *F*(*t*) > 0, where 2*t*_*d*_ is the touch duration and peak force *F*_max_ occurs at *t* = 0. Whisker oscillation and decay during slip-off events were fit by a Levenberg-Marquardt algorithm with 0.95 confidence intervals provided by the MATLAB *cftool* function.

### Numerical simulations of whisker dynamics during tactile exploration

The vibrational eigenmodes and eigenfrequencies of trimmed and full-size conical whiskers with fixed-base and free-tip boundary conditions were found in analytical form using the results of [54]. The found eigenfrequencies for the first six modes for full-size whisker are identical to published data [53]. To simulate whisker motion during the contact with pole, the eigenmodes and eigenfrequencies of a truncated and full-size conical whisker with an additional simple support at the pole position were used. The analytical calculations show that these eigenmodes depends strongly on the pole position along the whisker. After the whisker’s detachment from the pole, its motion was considered as a free vibration. All numerical simulations were performed in MATLAB. Time-dependent displacements of both forced and free periods of the whisker motion were calculated using sums of the first ∼100 eigenmodes to ensure full convergence.
